# Assessment of pain and functional outcomes after lower limb amputation: a scoping review

**DOI:** 10.1136/bmjopen-2025-110319

**Published:** 2026-03-10

**Authors:** Jin Min Kim, Shane J T Balthazaar, Khalid Alsayed, Thomas Nightingale, Deborah Falla, Sang-Hoon Yeo, Ziyun Ding

**Affiliations:** 1 School of Engineering, University of Birmingham, Birmingham, UK; 2 School of Sport, Exercise and Rehabilitation Sciences, University of Birmingham, Birmingham, UK; 3 Institute of Cardiovascular Sciences, University of Birmingham, Birmingham, UK; 4 International Collaboration on Repair Discoveries, The University of British Columbia, Vancouver, British Columbia, Canada; 5 Centre of Precision Rehabilitation for Spinal Pain (CPR Spine), School of Sport, Exercise and Rehabilitation Sciences, University of Birmingham, Birmingham, UK

**Keywords:** Gait, Amputation, Surgical, Chronic Pain, Lower Extremity

## Abstract

**Abstract:**

**Background:**

Pain, including phantom limb pain (PLP), residual limb pain (RLP) and low back pain (LBP), is highly prevalent after lower limb amputation (LLA) and compromises quality of life. Although both pain and function have been studied extensively, methods of assessment and reporting vary, limiting comparability. A clearer overview of how these domains are measured and interrelated is needed to guide research and practice.

**Objective:**

To synthesise evidence on how postamputation pain and functional outcomes have been assessed and reported in adults with LLA, and to examine reported relationships between pain and mobility/function.

**Design:**

Scoping review guided by Preferred Reporting Items for Systematic Reviews and Meta-Analyses extension for Scoping Reviews.

**Data sources:**

MEDLINE, Embase and PsycINFO (inception to 15 August 2025).

**Eligibility criteria:**

Quantitative studies that measured pain and functional outcome in adults with LLA.

**Data extraction and synthesis:**

Two reviewers independently extracted study characteristics, pain measures and functional outcomes in Covidence; findings were narratively synthesised.

**Results:**

Eighty-four studies were included. RLP (n=46), LBP (n=32) and PLP (n=28) were most frequently examined. Pain was mainly assessed by self-report scales; mobility was typically assessed by clinical tests and less often by biomechanical instrumentation. PLP was associated with altered gait and balance deficits; RLP with limited walking distance, asymmetric weight-bearing and reduced community participation; LBP with gait asymmetry, trunk–pelvis discoordination and increased energy cost of walking.

**Conclusions:**

Postamputation pain is often linked to reduced mobility and functional limitations. However, heterogeneous definitions and inconsistent methodology hinder synthesis across studies. Future research should combine validated pain scales with objective analysis, wearable sensors and musculoskeletal modelling to clarify mechanisms and inform rehabilitation.

**STRENGTHS AND LIMITATIONS OF THIS STUDY**

- This scoping review followed established methodological frameworks and Preferred Reporting Items for Systematic Reviews and Meta-Analyses extension for Scoping Reviews guidelines to ensure methodological rigour and transparent reporting.
- A comprehensive multidatabase search strategy was developed with an academic librarian.
- Study screening and data extraction were conducted independently by multiple reviewers.
- Heterogeneity in study design, pain assessment and functional outcomes precluded quantitative synthesis.
- No formal quality appraisal was undertaken, in line with scoping review methodology.

## Introduction

 Lower limb amputation (LLA), defined as the surgical removal of part or all of the lower extremity, is most commonly performed due to complications from peripheral vascular disease, diabetes or traumatic injury.^1^ Amputation levels vary from the toe or foot to transtibial (below-knee, BK) or transfemoral (above-knee, AK), each presenting a broad spectrum of physical and psychological challenges that can profoundly impact mobility and overall quality of life.^2 3^ LLA may be unilateral or bilateral, with bilateral cases often associated with greater mobility limitations and rehabilitation needs. In recent decades, the global incidence of LLA has increased, largely driven by ageing populations and the rising burden of non-communicable diseases such as diabetes and cardiovascular disease.^4^ Accordingly, rehabilitation following LLA has become a critical component of care, with a focus on improving function, promoting independence and managing secondary complications.^5 6^

Among the most common and debilitating complications are pain-related syndromes, including phantom limb pain (PLP), residual limb pain (RLP) and musculoskeletal pain.^7 8^ PLP refers to painful sensations perceived in the absent limb,^9^ often described as burning, stabbing or electric shock-like, and is believed to arise from maladaptive neural plasticity and central sensitisation.^10^ Epidemiological studies consistently indicate that PLP occurs with greater frequency in the lower limbs compared with the upper limbs.^11^ RLP, also referred to as ‘stump pain’, differs from PLP in that it originates from the actual tissue remaining after amputation.^12^ Patients frequently describe neuroma-related RLP as exhibiting hypersensitivity to pressure, light touch and thermal stimuli,^13^ with symptoms often manifesting in the early postoperative period and potentially subsiding over time.^14 15^ Low back pain (LBP) is another frequently reported secondary pain condition in individuals with LLA,^12^ predominantly stemming from gait alterations necessitated by prosthesis use, leading to increased biomechanical stress across the hip,^16^ lumbar spine^17^ and contralateral knee joint.^18^ Although LBP is not an amputation-specific pain per se, it is widely recognised as a secondary musculoskeletal condition commonly arising from compensatory gait mechanics following LLA and is therefore included in this review due to its relevance to functional mobility.

Previous narrative and systematic reviews have addressed postamputation pain across these different pain phenotypes, although with varying emphases.^15 19^ Across reviews focusing on PLP, RLP and LBP, a potential association between pain and functional limitations has been acknowledged, such as reduced prosthesis use,^15 20^ altered gait patterns^19 21^ or restricted participation in daily activities.^22^ However, despite these recurring observations, the relationship between postamputation pain and functional mobility has largely been discussed descriptively.^23 24^ To our knowledge, functional outcomes have not been systematically mapped or characterised in relation to pain severity, pain type or assessment approach in existing reviews, and findings across pain phenotypes remain dispersed across the literature. Consequently, the nature and extent of reported pain–function relationships following LLA remain fragmented and inconsistently described.

This fragmentation is also evident at the level of primary research, where pain and functional outcomes are frequently examined separately rather than within a unified analytical framework. Together with substantial heterogeneity in pain scales, functional tests and outcome definitions, this fragmentation complicates efforts to synthesise findings and establish a coherent evidence base on pain–function relationships following LLA.

Compounding this issue is the widespread reliance on subjective self-report measures in pain assessment. Most tools, such as the Visual Analogue Scale (VAS),^25^ Numeric Rating Scale (NRS)^26^ and the McGill Pain Questionnaire (MPQ),^27^ aim to quantify pain intensity at a specific moment, providing valuable insight into the patient’s perceived experience.^28^ However, these instruments are inherently limited in terms of reliability, interindividual comparability and objectivity, as they are influenced by psychological, emotional and contextual factors.^29^

Similarly, the assessment of functional status in individuals with LLA often relies on patient-reported measures or simple clinical tests, such as the 6-Minute Walk Test (6MWT), Timed Up and Go (TUG) and the Amputee Mobility Predictor (AMP).^30^ These tools are widely adopted due to their practicality and clinical utility in evaluating mobility and rehabilitation progress. However, while convenient, they may fall short in capturing the complex and nuanced ways in which different types and intensities of pain influence gait patterns and functional movement.

Despite these limitations, emerging evidence suggests that pain may have a measurable impact on various aspects of physical function in individuals with LLA. For example, Gailey *et al*^3^ found that slower walking speed during inpatient rehabilitation correlated with lower Medicare Functional Classification Levels (K-levels) and diminished motor capacity. Although pain was not directly measured, the authors suggested that discomfort and pain-related avoidance behaviour may contribute to impaired gait performance. Similarly, Seth *et al*^31^ reported a significant association between higher pain intensity and lower balance confidence, with increased fall risk in individuals with LLA. Nasri *et al*^32^ assessed interjoint coordination variability during gait in individuals with BK and proposed that factors such as residual limb discomfort and altered motor strategies may underlie diminished neuromuscular coordination. Taken together, these studies indicate that pain and functional performance in individuals with LLA are often examined within the same clinical context, although the nature of their relationship is explored indirectly and using heterogeneous approaches. Pain can restrict activity, while reduced function may, in turn, exacerbate pain through maladaptive movement patterns or compensatory mechanisms.^33^

Given this complex and potentially bidirectional relationship, there is a need to map and summarise existing literature to clarify how postamputation pain and functional mobility have been examined to date.

Accordingly, this scoping review aims to:

1. Review existing studies on postamputation pain and functional outcomes in individuals with LLA.
2. Summarise the assessment methods used for pain and function, along with key findings regarding their bidirectional relationship.
3. Identify research gaps and priorities for future investigation.

In undertaking this review, we acknowledge the substantial heterogeneity of study designs and assessment tools, and therefore adopted a scoping review methodology to systematically map the extent and nature of the existing evidence.

## Methods

### Study design

This scoping review was conducted in accordance with the five-stage methodological framework proposed by Arksey and O’Malley,^34^ with enhancements from Levac *et al*.^35^ Reporting followed the Preferred Reporting Items for Systematic Reviews and Meta-Analyses extension for Scoping Reviews (PRISMA-ScR) checklist.^36^ The review protocol was prospectively registered with the Open Science Framework (registration DOI: https://doi.org/10.17605/OSF.IO/4RG63). Patients and the public were not involved in the design, conduct, reporting or dissemination plans of our research. Accordingly, ethical approval was not sought, as the study was based exclusively on publicly available literature and did not involve human participants or identifiable personal data. Nevertheless, it forms part of a wider research project approved by the UK Health Research Authority and Health and Care Research Wales (Research Ethics Committee reference: 25/WM/0061, Integrated Research Application System project ID: 354627).

### Stage 1: identify the research question

This review was guided by the following research question: *‘What assessment methods have been used to evaluate postamputation pain and functional outcomes in individuals with LLA, and what findings have been reported regarding their bidirectional relationship?’*

### Stage 2: search strategy

A comprehensive search was conducted by the first author (JK) across three electronic databases: MEDLINE (via PubMed), Embase and PsycINFO, covering literature up to and including 15 August 2025. The search strategy was developed in collaboration with a health sciences librarian from the University of Birmingham, United Kingdom and was based on four core concepts:

1. *LLA* (amput*, transtibial, transfemoral).
2. *Pain* (pain*, PLP, RLP, LBP).
3. *Assessment or measurement* (assess*, scal*, tool*, measur*, evaluat*, test).
4. *Mobility or gait* (gait*, walk*, mobil*, ambulat*, biomech*, locomotion).

Additional studies were identified through reference list screening of relevant articles and reviews. A detailed example search strategy is available in online supplemental material.

### Data management

All retrieved records were imported into Covidence, a systematic review software platform. Duplicates were removed automatically and manually verified. Covidence was also used for title/abstract screening, full-text review, data extraction and resolving reviewer conflicts.

### Stage 3: study selection

**Figure 1:**
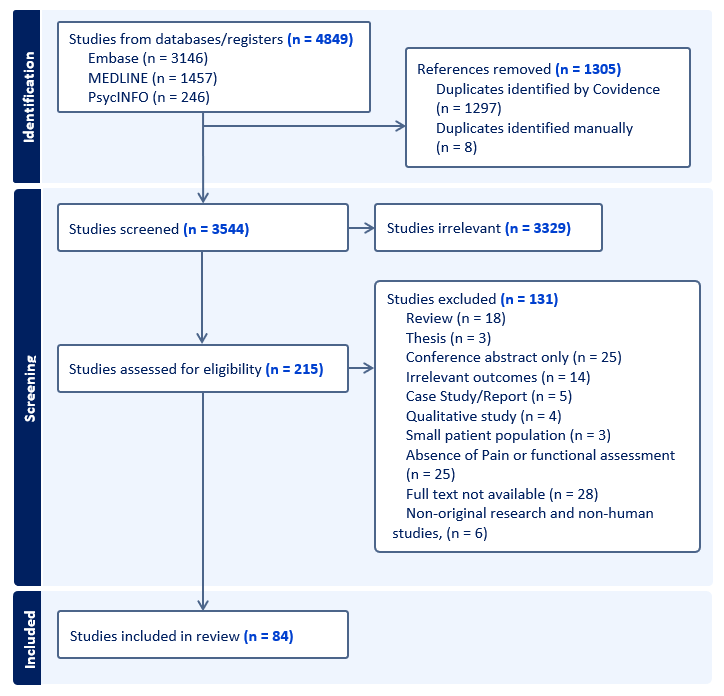
Preferred Reporting Items for Systematic Reviews and Meta-Analyses (PRISMA) flow diagram showing the identification, screening, eligibility and inclusion process of studies in this scoping review.

Two reviewers (JK, SB) independently assessed each title and abstract based on the prespecified eligibility criteria. Articles deemed irrelevant were excluded; those marked ‘include’ or ‘unclear’ proceeded to full-text assessment. The same reviewers independently retrieved and evaluated full-text articles to confirm their eligibility. Preprint articles, including those from platforms such as medRxiv, were also considered for inclusion if they met the eligibility criteria. Reasons for exclusion were recorded, including removal of review articles, conference abstracts without full text, theses or dissertations, qualitative studies and case reports. In line with commonly applied definitions, studies were also excluded if they had a very small sample size (<5 participants). Furthermore, studies were excluded if they did not report both pain and functional or mobility-related outcomes, or if the study population could not be clearly identified as individuals with LLA. For studies involving mixed samples, inclusion was only considered if outcomes for individuals with LLA were reported separately and both pain and function were explicitly evaluated; in such cases, at least half of the sample had to consist of individuals with LLA for the study to be retained. Discrepancies at any stage were discussed until consensus was reached. A PRISMA flow diagram was generated to illustrate the study selection pathway (see figure 1).

### Stage 4: charting the data

Data extraction was independently performed by two reviewers (JK, KA) using Covidence. Following this, JK conducted an additional manual full-text review of all included studies to ensure accuracy and consistency. The following information was extracted from each included study:

- *Study characteristics*: author(s), year of publication, country and study design.
- *Participant details*: sample size, mean age, biological sex distribution, level of amputation, height, weight, time since amputation and pain characteristics.
- *Assessment tools*: instruments used to evaluate pain (eg, VAS, MPQ) and function or mobility (eg, 6MWT, Berg Balance Scale, instrumented gait analysis).
- *Key findings*: reported pain intensity and frequency, gait or functional outcomes.
- *Study summary*: a brief synthesis of each study’s overall findings.

Any discrepancies in data extraction were resolved through discussion or by consulting a third reviewer (SB).

### Stage 5: data synthesis

Findings were synthesised narratively and summarised using descriptive statistics where appropriate. Results were organised according to participant characteristics, pain type and the pain and functional (mobility) assessment methods used. The frequency with which specific pain and functional assessment categories and associated functional outcomes were reported was also described.

Due to substantial heterogeneity in study designs, outcome measures and methodologies, no quantitative meta-analysis was conducted. Instead, patterns across studies were identified through narrative synthesis. In line with the Arksey and O’Malley framework^34^ and PRISMA-ScR guidelines, no formal critical appraisal was undertaken, as the aim of this scoping review was to map the breadth and nature of the existing evidence rather than to evaluate study quality.

## Results

**Figure 2:**
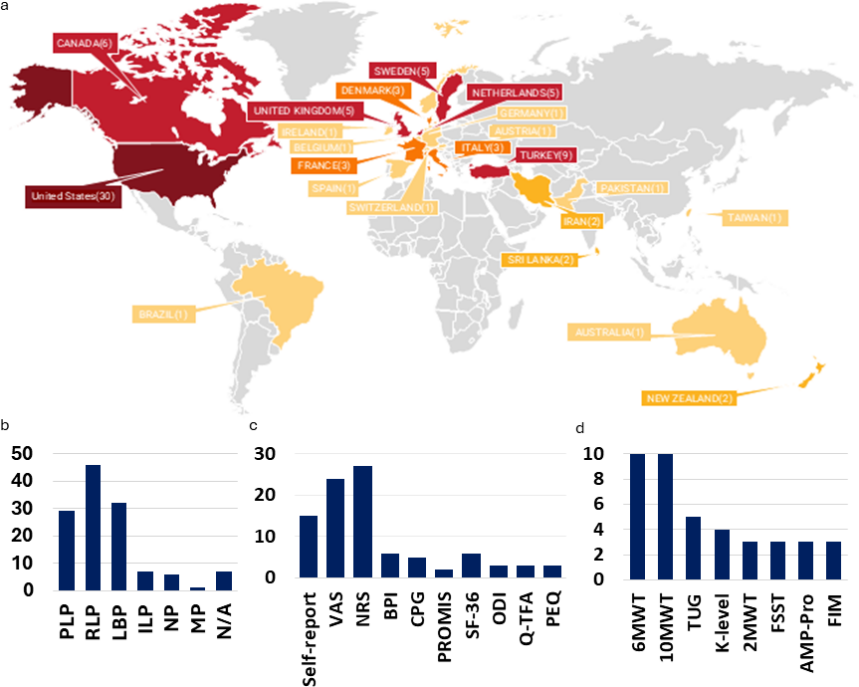
Overview of study characteristics across included studies. (a) Geographic distribution of the included studies, with the USA contributing the largest number (n=30), followed by Turkey (n=9), Canada (n=6), the United Kingdom (n=5), the Netherlands (n=5) and Sweden (n=5), with additional contributions from other countries. (b) Distribution of reported pain types among participants, including phantom limb pain (PLP), residual limb pain (RLP), low back pain (LBP), intact limb pain (ILP), neuropathic pain (NP) and musculoskeletal pain (MP). (c) Distribution of pain assessment instruments across included studies, showing that unidimensional pain intensity measures were most frequently used, whereas multidimensional and quality-of-life-related instruments were less commonly employed. Several studies reported pain presence using non-specified self-report formats. (d) Distribution of functional (mobility) assessment instruments reported more than once across the included studies. Clinical walking tests—particularly measures of walking endurance and gait speed—were most frequently used, while balance-focused tests, mobility classification systems and broader functional assessments were less commonly reported. BPI, Brief Pain Inventory; CPG, Chronic Pain Grade; FIM, Functional Independence Measure; FSST, Four-Square Step Test; 2MWT, Two-Minute Walk Test; 6MWT, Six-Minute Walk Test; 10MWT, Ten-Meter Walk Test; NRS, Numeric Rating Scale; ODI, Oswestry Disability Index; PEQ, Prosthesis Evaluation Questionnaire; PROMIS, Patient-Reported Outcomes Measurement Information System; Q-TFA, Questionnaire for Persons with a Transfemoral Amputation; SF-36, 36-Item Short Form Health Survey; TUG, Timed Up and Go; VAS, Visual Analogue Scale.

The database search yielded 4849 unique citations, of which 84 studies met the inclusion criteria following title/abstract and full-text screening (figure 1). The included studies spanned 24 countries, with the majority conducted in North America and Europe (figure 2a). Cross-sectional designs were most common (n=33), followed by cohort, longitudinal (n=19) and randomised controlled trial designs (n=15). Sample sizes varied widely, ranging from small exploratory studies^37^ to large registry-based (range 8–700) or multisite investigations.^38 39^

Participants represented a range of LLA levels. BK was most frequently reported,^231 37^,^91^ followed by AK, while through-knee and bilateral amputations were less commonly included. Reporting of participant characteristics such as age (approximately 20 to >70 years; mean typically 50–65 years), anthropometrics (height 160–180 cm; weight 60–90 kg), and time since amputation (ranging from several months to over 20 years) was heterogeneous across studies, limiting subgroup analyses. Detailed study and participant characteristics are summarised in online supplemental table 1.

### Pain types in LLA

Of the 84 included studies, 78 (92.8%) explicitly addressed pain, with many examining multiple pain types (figure 2b). RLP was the most frequently reported pain phenotype (45 studies),^3140^,^45 48^ followed by LBP (31 studies)^1617 31 37 40 41 46 47 54 58 65 68 71 74 80 82 88 92 93 95 97^,^107^ and PLP (28 studies).^4050 52 55^,^57 59 60 62 66 69 72 76 77 83 85 87^ Pain in the intact limb^31 41 65 75 93 95 106^ and neuropathic pain^40111^,^115^ were addressed far less frequently, while several studies assessed pain without specifying subtype.^38 53 73 78 90 116 117^

### Pain assessment tools

A wide range of pain assessment approaches was used (figure 2c). To characterise heterogeneity in pain assessment approaches, descriptive frequency statistics were used to summarise the distribution of pain measures across included studies. Fifteen studies relied on non-specified self-report formats capturing pain presence or broad severity categories. Among standardised tools, unidimensional pain intensity measures predominated, with the VAS^3741 43 46^,^48 58 64 67^ and NRS^3140 42 49 50 52^,^54 59 60 65 71 72 78^ used in over half of the included studies (n=51). Multidimensional pain instruments, including the MPQ,^80 110^ Brief Pain Inventory (BPI),^38 60 89 115 116^ Patient-Reported Outcomes Measurement Information System (PROMIS)^53 65 72 85 95^ and Chronic Pain Grade (CPG),^17 44 75 81 103^ were used less frequently (n=17).

Pain type-specific assessments, such as neuropathic pain screening tools (eg, Douleur Neuropathique 4)^40 112^ and condition-specific disability measures (eg, Oswestry Disability Index (ODI)),^58 100 107^ were applied in a small number of studies (n≤5), primarily in relation to LBP or neuropathic pain. A limited number of studies assessed joint-level pain using measures such as the Western Ontario and McMaster Universities Osteoarthritis Index (WOMAC) pain subscale^111^ or inferred pain-related functional impact from kinematic variables during gait.^102^ Pain was also evaluated indirectly through prosthesis-related mobility questionnaires (eg, Questionnaire for Persons with a Transfemoral Amputation,^95 96 106^ Prosthesis Evaluation Questionnaire (PEQ),^16 51 55^ Groningen Questionnaire Problems after Leg Amputation,^87 94^ and mobility classification systems such as the Daily Mobility Rating Classification^70 117^; n=10) and generic quality-of-life instruments (eg, 36-Item Short Form Health Survey (SF-36),^39 57 63 66 87 96^ EuroQol Five-Dimension Five-Level Questionnaire (EQ-5D-5L)^56^; n=7). Across all studies, pain assessment relied exclusively on subjective reporting, with no studies employing standardised experimental pain stimuli or objective physiological measures.

### Functional (mobility) and gait assessment tools

Functional mobility was most commonly assessed using clinical walking tests and patient-reported outcome (PRO) measures, with gait speed and endurance as the dominant domains (figure 2d). Across studies, gait was the primary focus of both clinical and instrumented assessments. Functional measures were grouped into clinical, instrumented and patient-reported domains.

Clinical assessments were dominated by walking-based tests, most frequently the 6MWT^41 42 70 71 81 86 90 93 102 104 117^ and Ten-Meter Walk Test (10MWT),^42 43 64 77 84 86 101 107 109^ with additional use of the TUG,^71 88 92 109^ Two-Minute Walk Test (2MWT)^78 88 92 109^ and Four-Square Step Test (FSST).^31 109^ Limb-loss-specific mobility measures included the AMP^91 97 115 117^ and K-level classification,^59 88 113^ while non-gait clinical tests such as the Functional Independence Measure (FIM)^73 110 116^ were used frequently.

Instrumented assessments primarily involved motion capture-based gait analyses examining joint kinematics and kinetics,^37 47 74^ supplemented by wearable sensors for spatiotemporal gait parameters and real-world walking activity.^77 90 105 109^ Non-gait instrumented assessments mainly focused on balance and postural control using force plates.^80 104 116^

Patient-reported outcomes (PROs) included measures of pain and disability (PROs-P&D) (CPG, BPI and ODI,^54 60 75 83 98^ prosthesis use and mobility (PROs-P&M) (Special Interest Group in Amputee Medicine mobility grades,^61 71 82^ Locomotor Capabilities Index,^56 113 115^ PEQ,^51 102^ Prosthetic Limb Users’ Survey of Mobility,^31 65^ and quality of life and participation (PROs-Q&P) (SF-36),^39 41 48 63 66 69 96 106 112^ WHO Quality of Life-Brief Version^59 94^ and EQ-5D-5L.^56109^,^111^

### Frequency of pain and functional assessment categories

**Figure 3:**
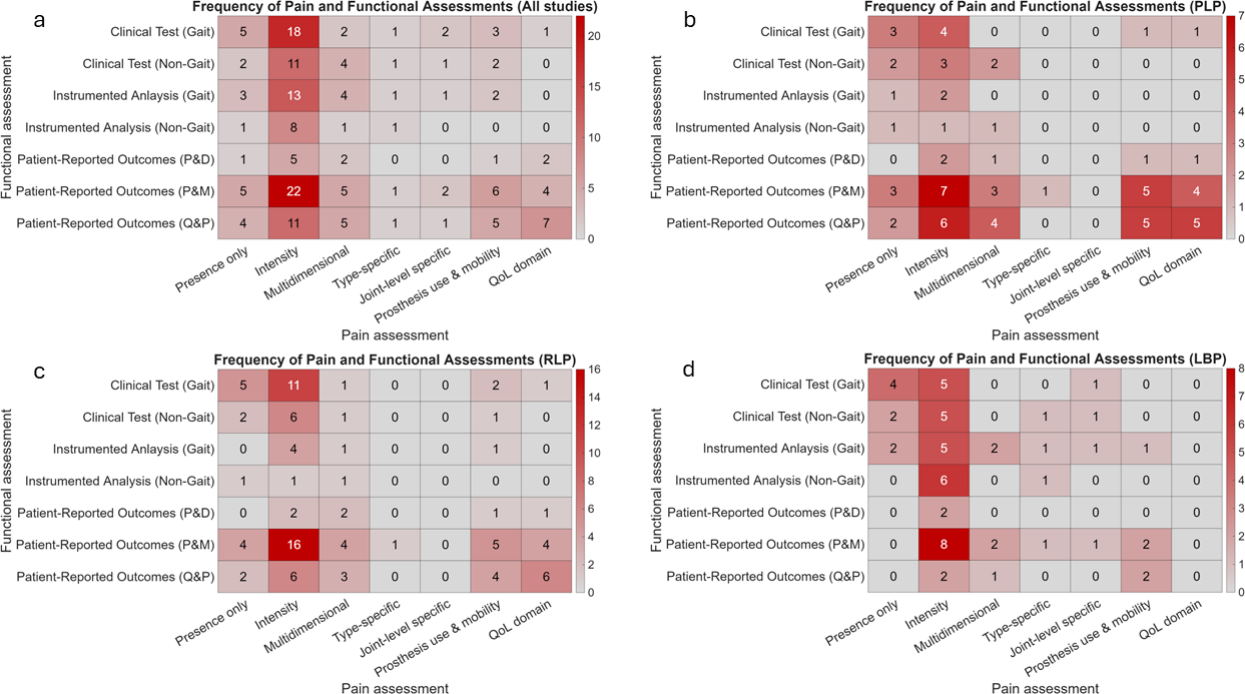
Frequency of pain and functional assessment categories across pain types. This figure presents heatmaps illustrating the frequency with which pain assessment categories were used in combination with functional assessment categories across the included studies. Panels show (a) all pain types combined, (b) PLP, (c) RLP and (d) LBP. Each cell represents the number of studies employing the corresponding pairing of pain and functional assessment categories, with darker shading indicating higher frequencies of co-occurrence. LBP, low back pain; P&D, pain and disability; PLP, phantom limb pain; P&M, prosthesis use and mobility; QoL, Quality of Life; Q&P, quality of life and participation; RLP, residual limb pain.

Across all pain types, pain intensity measures were most frequently paired with functional assessments, most commonly with PROs-P&M (up to n=22 studies), followed by clinical gait tests (n=18) and instrumented gait analyses (n=13) (figure 3a). In contrast, multidimensional, type-specific and joint-level pain assessments were rarely paired with functional measures across all pain categories (generally n≤2).

This pattern was consistent across pain phenotypes. In studies focusing on PLP, pain intensity measures were most frequently paired with PROs-P&M (n=7) (figure 3b). For RLP, the strongest co-occurrence was observed between pain intensity measures and PROs-P&M (n=16), followed by clinical gait tests (n=11) (figure 3c). In LBP-focused studies, pain intensity measures were most often combined with prosthesis-related outcomes (n=8) and clinical gait tests (n=5) (figure 3d).

### Pain types and associated functional outcomes

To synthesise movement-related findings, analyses were restricted to studies focusing on a single pain type, or those reporting pain-specific conclusions when multiple pain types were examined.

Despite its high prevalence, PLP was infrequently examined in relation to functional outcomes. Available evidence suggested potential associations with reduced prosthetic use^106^ and altered gait characteristics,^77^ particularly in relation to postural stability and sway control.

RLP was also commonly reported,^118 119^ yet relatively few studies examined its functional implications in detail. Reported associations included slower walking speed,^67^ reduced walking distance,^70 84^ impaired balance^64^ and reduced prosthetic use,^42^ as well as broader effects on physical activity^45 61^ and social participation.^56 75 95^

**Figure 4:**
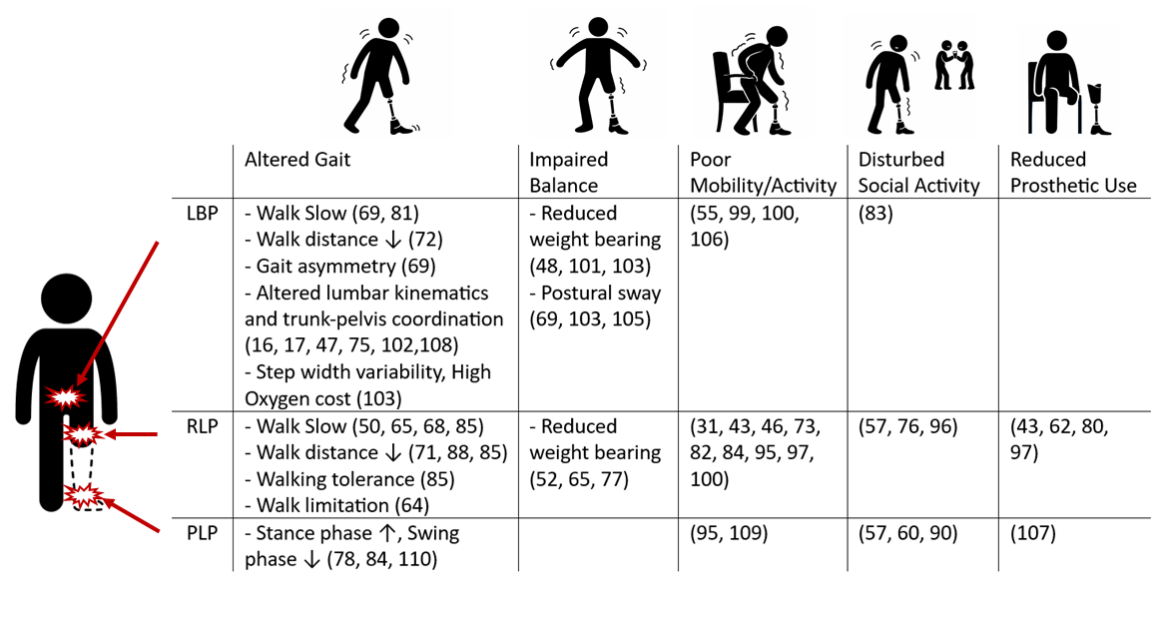
Cross-tabulated summary of pain-related functional impairments. This figure summarises reported associations between pain type and functional impairment domains in individuals with LLA. Bullet points indicate commonly reported outcomes, and numbers refer to citations. Icons represent functional domains, and red markers indicate pain location. Blank cells denote outcomes not reported in the literature. LBP, low back pain; LLA, lower limb amputation; PLP, phantom limb pain; RLP, residual limb pain.

Although LBP is reported with slightly lower prevalence than PLP and RLP,^118 119^ it has received disproportionate research attention regarding functional outcomes. Studies consistently reported gait alterations,^68^ trunk and pelvic motion abnormalities,^16^ balance impairments^68^ and reduced movement efficiency,^102^ highlighting a more extensive biomechanical characterisation of LBP compared with other pain types (figure 4).

Neuropathic pain was reported in a smaller subset of studies and was associated with reduced mobility efficiency.^115^ In contrast, musculoskeletal pain in the intact limb, despite its reported prevalence, was not examined as a distinct pain category in the included studies. Detailed study-level information on pain types, assessment tools and functional outcomes is provided in online supplemental table 2.

## Discussion

This scoping review mapped how postamputation pain and functional outcomes have been assessed and reported in adults with LLA, and summarised how studies have described relationships between these domains. Three overarching patterns emerged. First, pain measurement was dominated by unidimensional, intensity-based self-report tools, with relatively infrequent use of multidimensional instruments and a notable reliance on non-specified self-report formats. Second, functional outcomes were most commonly captured through gait-centric clinical tests and prosthesis- or mobility-related PRO measures, while instrumented biomechanical analyses were used less often. Third, when pain and function were examined together, pairings most frequently involved pain intensity measures with prosthesis-related PROs and walking tests; more detailed pain characterisation (eg, multidimensional or type-specific tools) was rarely linked to objective movement outcomes. Collectively, the literature documents that pain is common and function is often reduced after LLA, yet the prevailing assessment paradigm limits mechanistic interpretation and cross-study synthesis.

In line with the objectives of a scoping review, this study was not designed to formally test superiority between specific assessments or interventions. Rather, our aim was to map how postamputation pain and functional outcomes have been assessed and reported, and how the relationship between these domains has been described across the literature to date. Accordingly, the findings should be interpreted as an evidence map of current assessment approaches and reporting practices, rather than as comparative effectiveness conclusions.

Across the included literature, several common methodological characteristics were observed. Most studies employed cross-sectional designs with relatively small and heterogeneous samples, particularly in studies incorporating instrumented biomechanical analyses. These design features limit causal inference and the ability to examine how pain–function relationships evolve over time or in response to interventions. In addition, heterogeneity in outcome selection and limited integration of objective movement analysis further constrain mechanistic interpretation and reduce comparability across studies.

### Interpretation in the context of the broader pain–function literature

The separation of pain and function assessments identified in this review sits in contrast to established models in other clinical populations^120^,^125^ where pain–function relationships are commonly conceptualised as bidirectional and mediated by changes in motor control,^126^ protective movement behaviour,^127^ activity restriction,^128^ maladaptive loading^129^ and deconditioning.^130^ These frameworks are relevant to LLA because prosthesis use and gait compensation create clear pathways through which pain may influence movement, and through which altered movement may perpetuate pain. However, the present evidence base has not routinely operationalised these pathways using integrated measurement strategies. As a result, many studies describe associations between pain and mobility but do not enable inference about whether pain drives movement adaptations, whether compensations contribute to pain,^131^ or whether both evolve jointly over time.

### Assessment paradigm limitations

Two methodological tendencies appear to underpin this limitation. First, pain assessment relied exclusively on subjective reporting. While pain self-report is indispensable, the dominance of single-item intensity scales constrains insight into pain interference, temporal variability, sensory qualities and contextual triggers—dimensions that are particularly important in LLA where pain can be activity-dependent and prosthesis-related. The scarcity of multidimensional tools and the frequent use of non-specified pain formats further reduce comparability and may partially explain why findings across studies are difficult to reconcile, even when study designs appear similar.

Second, functional assessment was heavily weighted towards level-ground ambulation and clinically expedient mobility tests. Measures such as the 6MWT, 10MWT, TUG and mobility classification systems provide meaningful information about performance capacity, but they may not capture the task-dependent movement strategies through which pain may be expressed. Activities of daily living that may provoke or reveal pain-related adaptations—such as stair negotiation, uneven terrain walking, turning, transfers or dual-task balance—were systematically underrepresented.^132^ The predominance of cross-sectional designs further limits the ability to determine whether pain–function relationships are stable traits, fluctuate with rehabilitation stage and prosthesis fitting, or change in response to intervention.

### Biomechanical integration deficits

Although approximately 20 studies used motion capture and/or wearable sensors, biomechanical investigation was unevenly distributed across pain types. LBP received the most detailed movement-based characterisation, with multiple studies reporting trunk and pelvic kinematic alterations,^68 103^ reduced transverse plane motion^16^ and indicators of reduced movement efficiency. This is clinically plausible given the mechanical demands of prosthesis ambulation and compensatory strategies^133^ that may increase spinal loading; nevertheless, many biomechanical studies focused on isolated segmental metrics and rarely examined whole-body coordination, exposure to cumulative loading or longitudinal trajectories. Consequently, the field has not yet established whether observed gait patterns are antecedents of LBP, consequences of pain-related guarding or compensations driven by prosthetic-side deficits independent of pain.

In contrast, PLP and RLP—pain phenotypes frequently reported across the literature—were less often examined using objective movement analysis. For PLP, the literature strongly supports neuroplastic^134^ and sensorimotor mechanisms,^135 136^ but comparatively few studies have translated these mechanisms into measurable functional consequences. Evidence suggesting associations with balance deficits and altered gait exists, yet the link between PLP characteristics (eg, intensity, interference, episodic patterns) and task-level motor control remains insufficiently characterised. For RLP, plausible mechanical pathways—socket fit,^1^ local tissue irritation,^137^ loading intolerance^3^ and interface pressure—suggest direct effects on stance time,^138^ loading symmetry^139^ and terminal stance avoidance.^140^ However, few studies quantify these adaptations using integrated socket–residual limb measures, biomechanics and pain reporting, limiting the ability to move from description to mechanism-informed intervention.

Overall, the imbalance across pain types implies that research emphasis has been partly driven by what is easiest to capture biomechanically (eg, trunk and pelvic metrics in LBP) rather than by the prevalence and clinical burden of amputation-specific pain phenotypes. This is a central gap for the field: the most common pain presentations are not yet the most mechanistically interrogated.

### Methodological standardisation issues

Methodological inconsistency in instrumented studies remains a key barrier to synthesis and reproducibility. In particular, variable adherence to International Society of Biomechanics (ISB) recommendations—including inconsistent reporting of segment coordinate systems, joint coordinate system definitions and rotation sequence conventions—limits comparability of kinematic and kinetic outcomes across laboratories.^141 142^ This is especially consequential in LLA research, where prosthetic components, marker placement constraints (eg, socket-mounted markers) and residual limb segment definitions can substantially influence estimates of hip, pelvis and trunk motion. To improve reproducibility, future studies should report (at minimum) the marker set and prosthesis marker strategy, segment coordinate system definitions, joint rotation sequence, model scaling approach, filtering parameters and the exact operational definitions of key gait events and symmetry metrics. Adoption of an ISB-aligned reporting checklist would facilitate cross-study comparison and enable secondary synthesis in a field characterised by heterogeneous samples and small instrumented cohorts. Where feasible, the use of shared or openly available datasets may further enhance reproducibility and cross-study comparability, particularly in a research area characterised by small and heterogeneous instrumented samples.

### Clinical implications and future directions

Across pain phenotypes, the available evidence suggests that postamputation pain is associated with functional limitations across multiple domains. PLP and RLP, despite receiving less detailed biomechanical investigation, were consistently linked to reduced prosthetic use, slower walking speed, impaired balance and restrictions in daily activity and participation. In contrast, LBP has been examined more extensively and demonstrates consistent associations with altered gait patterns, trunk and pelvic motion abnormalities, balance impairments and reduced movement efficiency. Collectively, these patterns indicate that pain is not merely a coexisting symptom but appears to exert a measurable influence on functional performance after LLA. However, the current evidence base remains insufficient to draw definitive conclusions regarding the direction, magnitude or clinical significance of these relationships. Heterogeneity in pain types, outcome selection and study design, together with limited longitudinal and mechanistic investigation, constrains interpretation and precludes firm causal inference.

To advance the field beyond descriptive associations and towards clinically actionable evidence, several priorities should be addressed. First, future studies should adopt integrated assessment protocols that combine validated pain measures, preferably multidimensional tools capturing pain intensity, interference and activity dependence, with objective functional evaluation. Such integration would enable more direct linkage between pain profiles and movement adaptations and support interpretation of whether observed changes represent clinically meaningful improvement from a patient perspective, rather than statistical change alone, including consideration of established minimal clinically important difference thresholds.^143 144^ Future research may also benefit from incorporating objective approaches to pain evaluation that are well established in other clinical populations but have not yet been applied in LLA research, including standardised laboratory-based experimental pain paradigms such as contact heat-evoked potentials and the cold pressor test,^145^ as complementary inputs to functional and biomechanical assessment.

Second, research should prioritise the identification and validation of biomechanical markers sensitive to pain-related movement adaptations and clinically meaningful change. Candidate markers include gait asymmetry, stance time and loading avoidance, mediolateral trunk motion and altered hip–pelvis coordination. Examining these markers in relation to pain dimensions beyond intensity alone may enhance their translational relevance for rehabilitation planning and outcome monitoring.

Third, improved methodological standardisation is essential to enhance reproducibility and cross-study synthesis. As discussed, consistent adoption of ISB-compliant approaches and transparent reporting of key biomechanical parameters would facilitate meaningful comparison across studies, particularly in a field characterised by small and heterogeneous instrumented cohorts. Finally, longitudinal and intervention-based study designs are needed to clarify causal relationships between pain and function. Prospective studies and targeted interventions, including prosthetic optimisation, gait retraining and pain management strategies, should incorporate harmonised pain and objective movement outcomes to quantify mechanisms of change and inform evidence-based clinical practice.

### Strengths and limitations of this scoping review

This review systematically mapped the breadth of quantitative evidence on pain and functional outcomes after LLA using a multidatabase search, duplicate screening and duplicate extraction procedures, providing a structured synthesis of a heterogeneous literature. Nonetheless, heterogeneity in study designs, participant characteristics and outcome measures precluded quantitative synthesis. Restriction to English-language publications may have excluded relevant studies. Finally, no formal risk-of-bias assessment was undertaken, consistent with scoping review objectives; therefore, conclusions relate to evidence mapping rather than certainty of effects.

Several sources of bias inherent to the included study designs should be acknowledged. Many studies relied on convenience samples and cross-sectional designs, introducing potential selection bias and limiting causal inference. Variability in rehabilitation approaches, prosthetic components and assessment protocols may also contribute to performance bias, while heterogeneity in outcome selection and reporting raises the possibility of outcome reporting bias. These factors should be considered when interpreting the findings of this review.

## Conclusions

The literature consistently indicates that postamputation pain is associated with functional limitations, but heterogeneity in definitions and assessment approaches limits synthesis and mechanistic understanding. Pain assessment remains dominated by intensity-based self-report, and functional outcomes are largely gait-centred, with relatively limited integration of pain characterisation and objective movement analysis, particularly for PLP and RLP. Future work should implement integrated, standardised and longitudinal approaches combining multidimensional pain tools with objective biomechanics, wearable monitoring and, where appropriate, musculoskeletal modelling. Such efforts will be essential to clarify pain–function mechanisms and support more targeted, evidence-informed rehabilitation and prosthetic interventions for individuals with LLA.

## Supplementary material

10.1136/bmjopen-2025-110319 online supplemental file 1

10.1136/bmjopen-2025-110319 online supplemental table 1

10.1136/bmjopen-2025-110319 online supplemental table 2

## Data Availability

All data relevant to the study are included in the article or uploaded as supplementary information.
