# Supplementary material for: Assessment of pain and functional outcomes after lower limb amputation: a scoping review
Source: BMJ Open. 2026 Mar 10;16(3):e110319. doi: 10.1136/bmjopen-2025-110319 (PMC12983873; doi:10.1136/bmjopen-2025-110319)
Supplement: online supplemental file 1 [file bmjopen-16-3-s001.docx]

Supplementary Material 1. Search strategy

As an example, the MEDLINE search strategy included the following syntax:

(amput* OR transtibial OR transfemoral) AND (pain* OR "phantom limb pain" OR "residual limb pain" OR "low back pain") AND (assess* OR measur* OR evaluat* OR tool* OR scale*) AND (gait* OR walk* OR mobil* OR ambulat*).
