# Supplementary material for: Assessment of pain and functional outcomes after lower limb amputation: a scoping review
Source: BMJ Open. 2026 Mar 10;16(3):e110319. doi: 10.1136/bmjopen-2025-110319 (PMC12983873; doi:10.1136/bmjopen-2025-110319)
Supplement: online supplemental table 1 [file bmjopen-16-3-s002.docx]

Supplementary Table 1. Detailed Characteristics of Included Studies and Participants

| **Author(s)/year** | **Country** | **Sample Size** | **Age (Mean ± SD, yr; range)** | **Sex (M/F)** | **Height (cm)** | **Weight (kg)** | **Amputation Level** | **Time since amputation** |
| --- | --- | --- | --- | --- | --- | --- | --- | --- |
| Persson 1982 | Sweden | 69 | 67 | N/A | N/A | 64 | TH, AK, TK, BK, TA | N/A |
| Key findings: Maximal end-weight varies by stump type, amputation level, pain presence, and body weight. Patients with stump pain tolerate less end-bearing, while phantom pain patients tolerate more. Findings support individualizing socket pressure distributions. | | | | | | | | |
| Helm 1986 | Denmark | 107 | 75 / range: 38-95 | 59 M / 48 F | N/A | N/A | AK, BK, Bilateral (AK or BK) | 1~5 yr |
| Key findings: Best prognosis observed in younger, preoperatively independent individuals undergoing unilateral BK amputation and with no pain. Pain and higher-level amputations reduced functional outcomes, but did not significantly affect social dependence. | | | | | | | | |
| Kelly 1998 | United States | 11 | 64.6 ± 10.9 | 9 M / 2 F | 177 ± 9 | 86.5 ± 14.2 | BK | N/A |
| Key findings: Pain intensity correlates with change in gait velocity, particularly above a threshold (33%). Gait changes are highly individual. Pain should be considered in gait assessment and rehabilitation, especially in cases of socket discomfort. | | | | | | | | |
| Hoogendoorn 2001 | Netherlands | 64 (43 reconstruction, 21 amputation) | Reconstruction: 39.9 ± 16.2; Amputation: 44.6 | Reconstruction: 33 M / 10 F; Amputation: 16 M / 5 F | N/A | N/A | BK, TK, AK | > 8 mo |
| Key findings: Despite higher impairment ratings in amputees (mean 73.5% vs 17.6% in reconstruction group), quality of life and function were similar. | | | | | | | | |
| Jones 2001 | Australia | 27 | 65.4 ± 16.6 | 21 M / 6 F | 164.5 ± 22.7 | N/A | BK (unilateral transtibial) | Mean 42 d (range: 16–319 d) |
| Key findings: Both static weight-bearing and walking velocity improve early in prosthetic rehab. Pain contributes indirectly to gait performance via its effect on weight-bearing. Static weight-bearing is a modifiable predictor and clinically useful target during early rehabilitation. | | | | | | | | |
| Van der Schans 2002 | Netherlands | 437 | 65 ± 15 | 310 M / 127 F | N/A | N/A | AK/TK (170), BK (267) | Mean 10 yr (range: 1–80 yr) |
| Key findings: Phantom pain significantly reduces quality of life across several domains, particularly emotional and pain-related roles. Walking distance and stump pain were the strongest predictors of health-related Quality of Life. | | | | | | | | |

Supplementary Table 1. Detailed Characteristics of Included Studies and Participants

| **Author(s)/year** | **Country** | **Sample Size** | **Age (Mean ± SD, yr; range)** | **Sex (M/F)** | **Height (cm)** | **Weight (kg)** | **Amputation Level** | **Time since amputation** |
| --- | --- | --- | --- | --- | --- | --- | --- | --- |
| Chou 2003 | Taiwan | 15 | 47.3 ± 6.2 | 15 M | 167.2 ± 5.2 | 61.3 ± 4.7 | BK (transtibial) | 7.3 ± 5.1 yr |
| Key findings: Increased walking speed led to increased range of motion and joint loading but did not increase interface pressure or significantly change pain. Multiple axis foot provided moderate shock absorption. Pressure distribution aligned with reported pain. | | | | | | | | |
| Rudy 2003 | United States | 62 (38 lower limb amputation, 24 paraplegia) | Pain: 41.2 ± 11.9, Pain-free: 40.1 ± 12.1 | Pain: 22 M / 9 F Pain-free: 21 M / 10 F | Pain: 176.5 ± 8.3, Pain-free: 172.8 ± 13.8 | Pain: 82.1 ± 18.6 Pain-free:  79.3 ± 19.3 | AK (5) BK (14) | Pain: 14.56 ± 10.93 yr, Pain-free: 14.71 ± 10.88 yr |
| Key findings: Chronic pain impaired performance (quantity) but not task mechanics (style). Self-efficacy, physical/emotional functioning were strongest performance predictors; pain cognitions (e.g., catastrophizing) had inverse effects. | | | | | | | | |
| Mackenzie 2004 | United States | 161 | 35.2 ± 13.3 | 135 M / 26 F | N/A | N/A | AK (34), TK (18), BK (109) | 3, 6, 12, 24 mo follow-up |
| Key findings: TK associated with significantly worse functional outcomes than BK or AK. Sickness Impact Profiles scores did not differ between BK and AK. | | | | | | | | |
| Williams 2004 | United States | 89 | 44.2 / range: 16–89 | 62 M / 27 F | N/A | N/A | AK (16), BK (59), TK | 1, 6, 12, 24 mo follow-up |
| Key findings: Quality (perceived) of social support, not quantity (integration), predicts better short-term outcomes post-amputation, particularly in mobility and meaningful activity. Interventions should target improving relationship quality post-surgery. | | | | | | | | |
| Berge 2005 | United States | 15 | 51 ± 9 | 15 M | 179 ± 6 | 92 ± 17 | BK | ≥2 yr |
| Key findings: Shock-absorbing pylon provided no significant advantage over rigid pylons in gait mechanics, shock absorption, activity levels, comfort, pain, or fatigue among unilateral transtibial amputees. The only significant difference was knee angle at initial contact, suggesting amputees can modulate stiffness to adjust to pylon compliance. | | | | | | | | |
| Friel 2005 | United States | 19 | 56.5 ± 14.3 | 16 M / 3 F | N/A | N/A | AK (8), BK (11) | 11.8 ± 9.3 yr |
| Key findings: Individuals with LLA and LBP had greater iliopsoas length, weaker back extensors, and lower endurance. These may contribute to LBP via altered mechanics or instability. Differences in strength/endurance observed between BK and AK. | | | | | | | | |
| Kulkarni 2005 | United Kingdom | 202 | 48 | 174 M / 28 F | N/A | N/A | AK (77), BK (115), Bilateral (AK or BK) | Mean 19 yr (range: 2–40 yr) |
| Key findings: Chronic LBP is highly prevalent and often coexists with stump and phantom pain. No link to degenerative changes (MRI). Pain likely of myofascial origin, linked to altered posture and load distribution. Early gait retraining is advised. | | | | | | | | |

Supplementary Table 1. Detailed Characteristics of Included Studies and Participants

| **Author(s)/year** | **Country** | **Sample Size** | **Age (Mean ± SD, yr; range)** | **Sex (M/F)** | **Height (cm)** | **Weight (kg)** | **Amputation Level** | **Time since amputation** |
| --- | --- | --- | --- | --- | --- | --- | --- | --- |
| Norvell 2005 | United States | 62 amputees, 94 nonamputees | Amputees: 63.0 ± 11.9;  Nonamputees: 65.1 ± 11.3 | Amputees: 62 M; Nonamputees: 94 M | Amputees: 180 ± 10; Nonamputees: 180 ± 10 | Amputees: 77.5 ± 14.6; Nonamputees: 72.7 ± 11.1 | AK (18), BK (44) | ≥5 yr |
| Key findings: Knee pain was more than twice as prevalent in amputees (especially AK) vs. nonamputees. AK had 3.3 times increased risk of contralateral knee pain. BK had decreased risk of pain in amputated limb. Increased loading on intact limb likely cause. | | | | | | | | |
| Graham 2006 | United Kingdom | 75 | 53.5 ± NA | 59 M / 16 F | N/A | N/A | AK (25), BK (16), Transradial (13) | up to 34 yr |
| Key findings: Psychological issues (44% with psychiatric caseness; 67% with PTSD symptoms) and stump pain remain prevalent decades post-trauma. Stump pain was significantly linked to poorer mobility and psychological well-being. | | | | | | | | |
| Aksnes 2008 | Norway and Sweden | 118 (67 limb-sparing,  51 amputations, 4 secondary amputations) | 31 ± NA | 65 M / 53 F | N/A | N/A | AK (34), BK (5), TH (10) | Mean 13 yr (range: 6–22 yr) |
| Key findings: Limb-sparing results in better physical function (via Musculoskeletal Tumour Society score), although quality of life scores (via SF-36 and Toronto Extremity Salvage Score) were similar. Functional impairment (mostly among amputees) significantly impacted overall well-being, job choices, and physical activity. Chronic muscle pain, amputation, and tumour location above the knee were key predictors of poor function. | | | | | | | | |
| Hagberg 2008 | Sweden | 18 | 45 ± NA / range: 22–62 | 8 M / 10 F | 171 (range: 155–194) | 77 (range: 47–115) | AK | Mean 15 yr  (range: 10 mo – 33 yr) |
| Key findings: Osseointegrated prostheses significantly improved general and amputation-specific quality of life in transfemoral amputees over a 2-year follow-up. Improvements were seen in pain, mobility, prosthesis use, and global health perception. | | | | | | | | |

Supplementary Table 1. Detailed Characteristics of Included Studies and Participants

| **Author(s)/year** | **Country** | **Sample Size** | **Age (Mean ± SD, yr; range)** | **Sex (M/F)** | **Height (cm)** | **Weight (kg)** | **Amputation Level** | **Time since amputation** |
| --- | --- | --- | --- | --- | --- | --- | --- | --- |
| Smith 2008 | Ireland | 107 | 51.1 ± 14.3 / range: 16–83 | 88 M / 19 F | N/A | N/A | AK (32), BK (57), TK (4), TH (2), TA (2), Bilateral (10) | 17 ± 14.6 yr |
| Key findings: Back pain and RLP are common in ambulatory LLAs. Both types of pain cause mild interference on average but can be severe in some. | | | | | | | | |
| Morgenroth 2009 | United States | 17 (9 with LBP, 8 without LBP) | LBP: 54.7 ± 11.3, without LBP: 47.5 ± 12.2 | N/A | N/A | N/A | AK | LBP: 25.7 ± 17.1 yr, without LBP: 21.0 ± 14.3 yr |
| Key findings: No significant relationship found between either static or dynamic LLD and LBP in AK. Challenges common clinical assumption that LLD contributes to LBP. Suggests LBP in AK is likely multifactorial. | | | | | | | | |
| Tekin 2009 | Turkey | 19 (10 BK, 9 salvage surgery patients) | Amputee: 27.7 ± 5.3, Salvage: 28.4 ± 4.2 | 19 M | Amputee: 173.3 ± 3.7, Salvage: 172.9 ± 7.5 | Amputee: 72.8 ± 4.2,  Salvage: 74.3 ± 11.1 | BK | Amputee: 50.3 ± 54.2 mo,  Salvage: 66.1 ± 49.6 mo |
| Key findings: Amputees reported better general health and vitality (SF-36), and less pain than salvage patients. Functional performance was similar across both groups, despite salvage patients having moderate ankle joint degeneration. | | | | | | | | |
| Morgenroth 2010 | United States | 17 (9 with LBP, 8 without LBP),  6 healthy controls | LBP: 54.7 ± 11.3, without LBP: 47.5 ± 12.2 | N/A | N/A | N/A | AK | LBP: 25.7 ± 17.1 yr, without LBP: 21.0 ± 14.3 yr |
| Key findings: Greater transverse plane lumbar spine motion during gait is associated with presence of LBP in AK. Excessive rotation may stress intervertebral discs and contribute to pain. Suggests biomechanical mechanisms behind LBP in this group. | | | | | | | | |
| Vincent 2010 | Canada | 10 | 71 ± NA / range: 51–83 | 8 M / 2 F | N/A | N/A | N/A | 8–18 mo post-discharge |
| Key findings: A model evaluating potential vs effective mobility alongside modulating factors is feasible and useful. Many seniors with LLAs show mismatches between lab-based capabilities and real-world mobility due to social and health barriers. | | | | | | | | |

Supplementary Table 1. Detailed Characteristics of Included Studies and Participants

| **Author(s)/year** | **Country** | **Sample Size** | **Age (Mean ± SD, yr; range)** | **Sex (M/F)** | **Height (cm)** | **Weight (kg)** | **Amputation Level** | **Time since amputation** |
| --- | --- | --- | --- | --- | --- | --- | --- | --- |
| Couture 2011 | Canada | 16 | Positive appraisal: 63.6 ± 13.9, Negative appraisal: 70.0 ± 13.3 | 9 M / 7 F | N/A | N/A | AK (4), BK (12) | During hospitalization, rehabilitation, and 2–3 mo post-discharge |
| Key findings: Majority of participants appraised the amputation as positive and reported benefits including less pain, improved social contact, fewer health concerns, and reduced demands from others. Positive appraisal was linked to better adjustment (e.g., body image, function). | | | | | | | | |
| Devan 2012 | New Zealand | 145 | 56.8 ± 14.6 / range: 18–93 | 120 M / 25 F | N/A | N/A | AK | 27.1 ± 16.1 yr (range: 1–66 yr) |
| Key findings: High prevalence of LBP among traumatic AK individuals. No direct link between overall physical activity levels and presence of LBP. However, physical activity is reduced in those reporting activity restriction due to LBP. | | | | | | | | |
| Fortington 2013 | Netherlands | 82 | 67.8 ± 13.0 | 55 M / 27 F | N/A | N/A | AK (30), BK (52), Knee disarticulation | Baseline, 6, 18 mo follow-up |
| Key findings: Health-related QoL improves significantly in most domains after LLA, especially within the first 6 months. Older age and higher amputation level are associated with poorer physical function. | | | | | | | | |
| Hagberg 2014 | Sweden, Norway, Spain | 39 | 44.0 ± 12.4 | 17 M / 22 F | N/A | N/A | AK | N/A |
| Key findings: Osseointegration improves prosthetic function, mobility, and energy efficiency, despite no change in phantom/back pain. | | | | | | | | |
| Roth 2014 | United States | 297 | 63.2 ± 13.3 | 176 M / 121 F | N/A | N/A | AK (19), BK (41), Bilateral (11), Foot-level (11) | 6 mo follow-up |
| Key findings: Postacute care at inpatient rehabilitation group leads to higher prosthesis use, greater satisfaction with gait, and less prosthesis-related pain compared to skilled nursing facility group. No significant difference between inpatient rehabilitation group and home in most outcomes. | | | | | | | | |

Supplementary Table 1. Detailed Characteristics of Included Studies and Participants

| **Author(s)/year** | **Country** | **Sample Size** | **Age (Mean ± SD, yr; range)** | **Sex (M/F)** | **Height (cm)** | **Weight (kg)** | **Amputation Level** | **Time since amputation** |
| --- | --- | --- | --- | --- | --- | --- | --- | --- |
| Russell Esposito 2014 | United States | 16 (9 with LBP, 7 without LBP),  12 healthy controls | LBP: 32.1 ± 5.2, without LBP: 28.4 ± 6.4,  Control: 25.1 ± 3.1 | 16M | LBP: 180 ± 6,  without LBP: 179 ± 7, Control: 179 ± 6 | LBP: 90.6 ± 10.9,  without LBP: 82.4 ± 8.0, Control: 79.5 ± 10.0 | AK | LBP: 23 ± 21 mo without LBP: 43 ± 20 mo |
| Key findings: LBP in AK is linked to altered frontal/sagittal trunk–pelvis coordination during gait, potentially compensating for loss of limb. Frontal coordination patterns shifted more with speed in LBP group. Transverse coordination unaffected. | | | | | | | | |
| Segal 2014 | United States | 10 | 56 ± 12 / range: 27–70 | 9 M / 1 F | 180 ± 10 | 88 ± 11 | BK | ≥2 yr |
| Key findings: Torsion adapters provide modest benefits in terms of daily activity at low-medium intensities and reduce perceived interference from residual limb pain. No effect on fatigue or straight-line walking distance (6MWT). | | | | | | | | |
| Ladlow 2015 | United Kingdom | 65 | 29 ± 6 / range: 20–52 | 63 M / 2 F | 179 ± 8 (range: 163–200) | 93 ± 17 (range: 64–135) | AK (23), Bilateral (23), Triple (8), AK-non-operational (11) | 34 ± 14 mo |
| Key findings: Despite high injury severity, military amputees achieved functional and mental health levels comparable to healthy adults. 95% were independent in ADL. | | | | | | | | |
| Soin 2015 | United States | 10 | 54 / range: 27-77 | 4 M / 6 F | N/A | N/A | AK (4), BK (6) | 7 yr (range: 0.8–18 yr) |
| Key findings: High-Frequency electrical nerve block is safe, feasible, and effective in reducing both phantom and stump pain. Pain relief extended beyond treatment window. Functional gains and reduced medication use support further large-scale trials. | | | | | | | | |

Supplementary Table 1. Detailed Characteristics of Included Studies and Participants

| **Author(s)/year** | **Country** | **Sample Size** | **Age (Mean ± SD, yr; range)** | **Sex (M/F)** | **Height (cm)** | **Weight (kg)** | **Amputation Level** | **Time since amputation** |
| --- | --- | --- | --- | --- | --- | --- | --- | --- |
| Anaforoglu 2016 | Turkey | 40 | Group 1: 38.0 ± 10.8,  Control: 36.0 ± 10.3 | 40 M | Group 1: 171.0 ± 5.2,  Control: 172.0 ± 5.5 | Group 1: 71.0 ± 9.7 Control: 73.0 ± 8.3 | AK | Group 1: 16.4 ± 13.5 yr, Control: 13.4 ± 10.0 yr |
| Key findings: The back school program combined with an exercise program significantly decreased pain intensity, reduced disability, and improved spinal flexibility among transfemoral amputees with mechanical LBP compared to controls. | | | | | | | | |
| Fatone 2016 | United States | 23 (12 with LBP, 11 without LBP) | LBP: 47.5 ± 12.6, without LBP: 48.3 ± 13.5 | LBP: 9 M / 3 F, without LBP: 10 M / 1 F | LBP: 179 ± 7, without LBP: 179 ± 6 | LBP: 79.1 ± 13.5, without LBP: 83.0 ± 13.5 | AK | LBP: 16.3 ± 12.7 yr, without LBP: 14.7 ± 12.8 yr |
| Key findings: Although differing lumbar spine motion patterns were observed between AK individuals with and without LBP, these patterns were not independently associated with LBP status. Suggests mild LBP may not be linked to spinal kinematics. | | | | | | | | |
| Ladlow 2016 | United Kingdom | 100 | 29 ± 6 | ≥ 95 M | Immediate BK: 180 ± 9, Delayed BK: 178 ± 9, Immediate AK: 182 ± 9, Limb salvage: 177 ± 8 | N/A | AK (10), immediate BK (11), delayed BK (15), Bilateral (43) | Delayed BK : 10 ± 11 mo |
| Key findings: Although differing lumbar spine motion patterns were observed between AK individuals with and without LBP, these patterns were not independently associated with LBP status. Suggests mild LBP may not be linked to spinal kinematics. | | | | | | | | |
| Mackenzie 2016 | Canada | 190 | 63.5 ± 14.2 | 136 M / 54 F | N/A | N/A | AK (32), BK (131), Bilateral BK (19), Complex cases (8) | N/A |
| Key findings: Maximum walk test is a feasible test for most lower limb amputees in inpatient rehab. Fatigue, pain, and use of gait aids were key barriers to administration and performance. Data can guide targeted interventions to improve discharge mobility and data collection. | | | | | | | | |

Supplementary Table 1. Detailed Characteristics of Included Studies and Participants

| **Author(s)/year** | **Country** | **Sample Size** | **Age (Mean ± SD, yr; range)** | **Sex (M/F)** | **Height (cm)** | **Weight (kg)** | **Amputation Level** | **Time since amputation** |
| --- | --- | --- | --- | --- | --- | --- | --- | --- |
| Devan 2017 | New Zealand | 208 | 52 ± 9 | 154 M / 54 F | N/A | N/A | AK (78), BK (130) | 21 ± 13 yr |
| Key findings: LBP in people with nondysvascular LLA is influenced by phantom pain, residual-limb issues, and multiple comorbidities. LBP intensity is also impacted by pain-provoking tasks like sit-to-stand. Employment appears protective. | | | | | | | | |
| George 2017 | United States | 53 | 65.2 ± 10.7 | 24 M / 29 F | N/A | N/A | AK | 29 ± 18 mo |
| Key findings: AK did not worsen ambulation in most cases. Male sex, better pre-op function, and absence of persistent phantom pain predicted better ambulation. Amputation improved physical QoL scores (VR-12), highlighting importance of phantom pain control. | | | | | | | | |
| Mahon 2017 | United States | 67 amputees, 76 healthy controls | 28.5 ± 6.4 | Amputees: 67 M, Controls: 76 M | 180 ± 6 | 90.2 ± 12.9 | AK | 2.5 ± 1.9 yr |
| Key findings: While young, active AK amputees showed biomechanical and functional deficits versus controls, impairments were less severe than in more diverse or older groups. Data can serve as clinical benchmarks. Longitudinal tracking needed for long-term effects. | | | | | | | | |
| Atic 2018 | Turkey | 126 | Vascular: 56.5 ± 6.9, Traumatic: 37.3 ± 10.1, Landmine: 36.2 ± 11.5 | 80 M / 46 F | ~170 | Vascular: 78.4 ± 6.1, Traumatic: 75.0 ± 8.3, Landmine: 73.5 ± 8.8 | AK (21), BK (105) | ≥6 mo |
| Key findings: Functional status, daily living activities, and quality of life were significantly lower in vascular amputees compared to trauma and landmine amputees. Trauma and landmine amputees showed similar outcomes across most parameters except emotional role and mental health, suggesting these two groups can be grouped together for practical purposes. | | | | | | | | |
| Aydin 2018 | Turkey | 88 (44 with test sockets;  44 without test sockets) | Test socket group: 40.3 ± 12.1, Without test socket group: 38.9 ± 11.5 | Test socket: 28 M / 16 F, Without test socket: 26 M / 18 F | Test socket: 172.2 ± 5.6, Without test socket: 170.2 ± 6.1 | Test socket: 77.1 ± 8.5, Without test socket: 75.8 ± 8.7 | Test socket: AK (12), BK (32) Without test socket: AK (13), BK (31) | ≥6 mo |
| Key findings: Use of a test socket significantly improved prosthetic satisfaction, reduced pain during walking, and improved functional outcomes for both transtibial and transfemoral amputees. Despite increased initial prosthetic cost, long-term advantages include fewer complaints and better functionality. | | | | | | | | |

Supplementary Table 1. Detailed Characteristics of Included Studies and Participants

| **Author(s)/year** | **Country** | **Sample Size** | **Age (Mean ± SD, yr; range)** | **Sex (M/F)** | **Height (cm)** | **Weight (kg)** | **Amputation Level** | **Time since amputation** |
| --- | --- | --- | --- | --- | --- | --- | --- | --- |
| Esfandiari 2018 | Iran | 587 | 43 ± 6.5 | 587 M | N/A | N/A | TH (29), AK (458), TK (94) | 22 ± 4 yr |
| Key findings: Patients with higher-level amputations (TH, AK) had more pain and lower function than those with TK. Pain persisted even decades post-amputation. Findings highlight need for lifelong support and tailored rehab. | | | | | | | | |
| Golyski 2018 | United States | 8 amputees, 5 healthy controls | AK: 23–34, BK: 24–45, Control: 20–31 | N/A | AK: ~170–190 BK: ~180–190 Control: ~170–190 | AK: 71.4–101.2 BK: 90.9–135.6 Control: 61.5–105.7 | AK (3), BK (4), TK (1) | ~5.5 to 133 mo |
| Key findings: Persons with LLA exhibit task-specific compensations during turns—especially in frontal and sagittal trunk/pelvic motion. These adaptations may increase long-term LBP risk through altered mechanics and loading patterns. | | | | | | | | |
| Allami 2019 | Iran | 247 | 52.4 ± 7.4 | 245 M / 2 F | N/A | N/A | BK | 29.8 ± 6.2 yr |
| Key findings: High prevalence of musculoskeletal pain (stump, phantom, low back) among veterans with below-knee amputation. Pain types are interrelated, emphasizing the importance of comprehensive and regular pain assessments for improved quality of life and rehabilitation outcomes. | | | | | | | | |
| Butowicz 2019 | United States | 32 (19 with LBP; 13 without LBP) | LBP: 35.8 ± 7.3, without LBP: 33.1 ± 7.5 | 32 M | LBP: 179.6 ± 7.2,  without LBP: 178.6 ± 4.8 | LBP: 91.9 ± 15.8, without LBP: 89.5 ± 14.8 | LBP: AK (8), BK (11) without LBP: AK (2), BK (11) | LBP: 8.7 ± 4.1 yr, without LBP: 6.1 ± 4.2 yr |
| Key findings: Persons with lower-limb loss and chronic low back pain exhibited impaired trunk postural control during unstable sitting compared to amputees without pain. These deficits might contribute to the persistence of LBP due to altered neuromuscular control and increased spinal instability, suggesting a biomechanical component to chronic low back pain in amputees. | | | | | | | | |
| Caliskan Uckun 2019 | Turkey | 51 amputees, 51 healthy controls | PIN/LOCK: 50.3 ± 13.7, VASS: 44.8 ± 12.6, Control: 44.4 ± 11.4 | Amputees: 45 M / 6 F | N/A | N/A | BK | PIN/LOCK: 22.6 ± 13.6 yr, VASS: 16.4 ± 13.3 yr |
| Key findings: Physical activity and QoL were significantly lower in people with amputation compared to controls. There was no significant difference between VASS and PIN/LOCK users in terms of physical activity or QoL. PIN/LOCK users’ scores were closer to controls. | | | | | | | | |

Supplementary Table 1. Detailed Characteristics of Included Studies and Participants

| **Author(s)/year** | **Country** | **Sample Size** | **Age (Mean ± SD, yr; range)** | **Sex (M/F)** | **Height (cm)** | **Weight (kg)** | **Amputation Level** | **Time since amputation** |
| --- | --- | --- | --- | --- | --- | --- | --- | --- |
| Dakhil 2019 | France and China | 8 | 43.6 ± 9.9 | 8 M | 172 ± 11 | 67.3 ± 11.4 | BK | ≥6 mo |
| Key findings: Pressure measurement, especially static pressure at the proximal posterior area of the stump, is a relevant factor for assessing prosthesis adequacy and user satisfaction. A threshold of <0.9 kPa/kg may help ensure long-term satisfaction. | | | | | | | | |
| Facione 2019 | France | 12 (5 with LBP, 7 without LBP) | Total: 39 ± 11.8, LBP: 43 ± 13.7, without LBP: 37 ± 10.6 | 10 M / 2 F | N/A | N/A | AK | LBP: 10 ± 11.9yr, without LBP: 11 ± 9.7yr |
| Key findings: AK frequently present sagittal imbalance. Decreased thoracic kyphosis appears to be an effective compensatory mechanism in asymptomatic AK. Cervical compensation and postural misalignment may contribute to LBP. | | | | | | | | |
| Gilmore 2019 | United States | 26 (12 Peripheral Nerve Stimulation group,  14 placebo group) | Overall: 46.5 ± 12.7,  PNS: 48.3 ± 12.3, Placebo: 45.0 ± 13.2 | 20 M / 6 F | N/A | N/A | AK (15), BK (11) | Overall: 7.0 ± 6.6 yr, PNS: 6.4 ± 4.6 yr, Placebo: 7.5 ± 8.1 yr |
| Key findings: Percutaneous peripheral nerve stimulation is safe and provides sustained, clinically significant pain relief and reduced pain interference in chronic postamputation pain. Benefits persisted up to 12 months post-therapy in the majority of patients. | | | | | | | | |
| Kulunkoglu 2019 | Turkey | 40 (20 Mirror Therapy-MT group,  20 Phantom exercise-PE group) | MT group: 32.6 ± 7.4, PE group: 29.6 ± 6.9 | MT group: 12 M / 8 F, PE group: 13 M / 7 F | MT group: 167.7 ± 6.8, PE group: 170.1 ± 6.2 | MT group: 67.1 ± 9.7, PE group: 68.1 ± 11.6 | BK | MT: median 13 mo  PE: median 13.5 mo |
| Key findings: Both MT and PE significantly reduced phantom limb pain and improved quality of life and psychological status, with MT showing significantly greater efficacy. Study supports MT as a highly effective, simple, and low-cost treatment for phantom limb pain. | | | | | | | | |

Supplementary Table 1. Detailed Characteristics of Included Studies and Participants

| **Author(s)/year** | **Country** | **Sample Size** | **Age (Mean ± SD, yr; range)** | **Sex (M/F)** | **Height (cm)** | **Weight (kg)** | **Amputation Level** | **Time since amputation** |
| --- | --- | --- | --- | --- | --- | --- | --- | --- |
| Leijendekkers 2019 | Netherlands | 40 | AK: median 56 / IQR: 45–59 BK: median 43 / IQR: 29.5–57.5 | 22 M / 18 F | N/A | N/A | AK (31), BK (9) | median 8 yr (IQR: 3.0–19.8) |
| Key findings: Osseointegration significantly improved function, Health-related QoL, and prosthetic satisfaction. Back pain didn’t change overall but improved in mobile patients. | | | | | | | | |
| Spahn 2019 | United States | 9 | 29 ± 6 / range: 23–39 | 8 M / 1 F | N/A | N/A | BK | 13 ± 3 mo |
| Key findings: Late BK led to reduced pain, improved gait parameters, and greater functional mobility (running, long-distance walking, no assistive devices). Despite residual asymmetries, overall mobility improved significantly post-BK. | | | | | | | | |
| Geertzen 2020 | Netherlands | 48 | At time of amputation: 41 / IQR: 28.5–46.0 | 8 M / 40 F | N/A | N/A | AK (9), TK (18), BK (16) | median 5.5 yr (IQR: 3.0–11.0) |
| Key findings: Amputation led to clinically meaningful pain and mobility improvements in most patients with Complex Regional Pain Syndrome Type I. Some recurrence of Complex Regional Pain Syndrome Type I occurred (confirmed in 4/47). Deterioration in intimacy/self-confidence was reported in ~25% of participants. | | | | | | | | |
| Mahon 2020 | United States | 32 | AK: 28.1 ± 6.2,  BK: 26.7 ± 6.0 | 32 M | AK: 177.9 ± 7.8, BK: 178.5 ± 4.0 | AK: 83.8 ± 10.8, BK: 84.1 ± 10.9 | AK (10), BK (22) | 0, 2, 4, 6, and 12 mo follow-up |
| Key findings: Trunk-pelvis motion patterns adapt significantly within the first year post-amputation. For BK amputees, LBP increased over time despite more stable kinematics. For AK group, reduced LBP aligned with improved coordination and decreased motion. | | | | | | | | |
| Pleus 2020 | Switzerland | Receptive field phase: 31;  Gait stimulation: 18 | Receptive: 56.8 ± 14.6, Gait: 59.1 ± 14.1 | N/A | Receptive: 177.4 ± 7.7, Gait: 178.7 ± 6.0 | Receptive: 88.2 ± 20.2, Gait: 88.4 ± 11.4 | BK | Receptive: 11.7 ± 12.3 yr, Gait: 10.8 ± 10.7 yr |
| Key findings: Receptive fields are stable over time and viable targets for repeated electrical stimulation. However, acute electrical stimulation does not significantly impact gait or pain. Chronic use should be tested for potential therapeutic effects. | | | | | | | | |

Supplementary Table 1. Detailed Characteristics of Included Studies and Participants

| **Author(s)/year** | **Country** | **Sample Size** | **Age (Mean ± SD, yr; range)** | **Sex (M/F)** | **Height (cm)** | **Weight (kg)** | **Amputation Level** | **Time since amputation** |
| --- | --- | --- | --- | --- | --- | --- | --- | --- |
| Damiani 2021 | Italy | 302 | 60.6 ± 14.2 | 240 M / 62 F | N/A | N/A | AK (148), BK (154) | 2.04 ± 1.68 yr |
| Key findings: Regular prosthesis use and BK significantly improve likelihood of achieving community ambulation. Pain intensity and amputation side had no significant impact. Age was a negative predictor. Suggests tailoring rehab based on predictive factors. | | | | | | | | |
| Fernandes 2021 | Denmark | 30 amputees, 30 healthy controls | 51 ± 18 | Amputees: 16 M / 14 F, Controls: 16 M / 14 F | N/A | N/A | AK - proximal (12), AK - distal (14), BK (4) | 7 ± 3 yr (range: 2–12 yr) |
| Key findings: Patients with tumor prostheses exhibit significant long-term impairments in strength, mobility, and QoL. Pain and muscle weakness are key factors affecting QoL. Objective functional assessments should guide rehab and patient education pre-op. | | | | | | | | |
| Linden 2021 | Belgium | 16 | Median 55.5 | 9 M / 7 F | N/A | N/A | AK (12), BK (4) | ≥1 yr |
| Key findings: Most patients were satisfied with the amputation (81%), and 69% reported improved pain and mobility. PLP persisted in most but was usually mild. Outcomes support amputation as a potential option in well-screened chronic pain cases. | | | | | | | | |
| METRC 2021 | United States | 639 (488 limb salvage; 151 amputation) | Mean 38.4 ± 12.3 | Amputees: 124 M / 27 F | N/A | N/A | BK | 18 mo |
| Key findings: For severe distal tibial, ankle, and mid/hindfoot injuries, amputation resulted in better self-reported mobility but similar pain severity, participation in vigorous activities, and work return compared to limb salvage. Amputation considered a viable option, particularly for complex injuries requiring flaps or with severe bone loss. Long-term outcomes were poor in both groups, highlighting the complexity of managing severe lower-limb trauma. | | | | | | | | |
| Srinivasan 2021 | United States | 22 (15 with AMI BKA,  7 with traditional BKA) | AMI: 39.0 ± 12.1, Traditional: 45.5 ± 16.9 | AMI: 9 M / 6 F Traditional: 3 M / 4 F | N/A | N/A | BK | N/A |
| Key findings: agonist–antagonist myoneural interfaces (AMI) enables more precise and differentiated motor control, improved proprioception, greater phantom ROM, and reduced pain vs traditional BK. AMI users may have enhanced myoelectric prosthesis control and better functional outcomes. | | | | | | | | |

Supplementary Table 1. Detailed Characteristics of Included Studies and Participants

| **Author(s)/year** | **Country** | **Sample Size** | **Age (Mean ± SD, yr; range)** | **Sex (M/F)** | **Height (cm)** | **Weight (kg)** | **Amputation Level** | **Time since amputation** |
| --- | --- | --- | --- | --- | --- | --- | --- | --- |
| Trouillez 2021 | France | 43 (20 AK, 23 arthrodesis) | AK: 73.8 ± 13.8, Arthrodesis: 77.7 ± 11.6 | AK: 10 M / 10 F, Arthrodesis: 7 M / 16 F | N/A | N/A | AK | AK: 4.2 ± 4.0 yr, Arthrodesis: 9.7 ± 4.7 yr |
| Key findings: AK patients had comparable infection control but significantly better pain relief, mobility, and quality of life than arthrodesis patients. AK should not be viewed as a last resort, but a valid early alternative in severe cases. | | | | | | | | |
| Younesian 2021 | Canada | 11 | 57.91 ± 15.63 | 6 M / 5 F | N/A | N/A | AK (4), BK (7) | ≥6 wk |
| Key findings: The 6MWT induces physical and pain-related fatigue in LLAs, reflected by HR rise, decreased cadence/speed, and increased stance ratio. IMU + HR monitoring during 6MWT offers valuable clinical insights for fatigue-related interventions. | | | | | | | | |
| Zaheer 2021 | Pakistan | 24 (12 experimental, 12 control) | Experimental: 45.3 ± 11.1, Control: 40.5 ± 12.5 | 17 M / 7 F | N/A | N/A | AK, BK | within 2 yr |
| Key findings: Phantom exercises added to mirror therapy and routine physiotherapy significantly improved pain but showed no added benefit in mobility or overall quality of life (except in bodily pain domain of SF-36). | | | | | | | | |
| Atar 2022 | Turkey | 42 (21 with NAA;  21 with AHA) | NAA: 37.0 ± 10.0, AHA: 40.5 ± 7.6 | 42 M | N/A | N/A | BK | NAA: 151.5 ± 126.2 mo, AHA: 188.6 ± 109.8 mo |
| Key findings: No significant differences found between NAA and AHA prosthetic feet in terms of functional capacity, pain severity, satisfaction level, and quality of life, except descending ramps difficulty, which favoured the AHA group. Reasons for dissatisfaction included inflexibility (NAA group) and frequent dysfunction of hydraulic ankle (AHA group). | | | | | | | | |
| Deldar 2022 | United States | 146 | 61.7 ± 12.3 | 102 M / 44 F | N/A | N/A | TM and BK | 23.2 mo (range: 0.7–97.6 mo) |
| Key findings: TM amputation has variable healing outcomes; patients requiring higher-level amputation had more complications and worse functional scores. Post-op infection significantly increases risk of TMA failure. Function and mobility can still be preserved in many cases. | | | | | | | | |

NAA: Non-Articulated Ankles; AHA: Articulating Hydraulic Ankles; TM: Transmetatarsal

Supplementary Table 1. Detailed Characteristics of Included Studies and Participants

| **Author(s)/year** | **Country** | **Sample Size** | **Age (Mean ± SD, yr; range)** | **Sex (M/F)** | **Height (cm)** | **Weight (kg)** | **Amputation Level** | **Time since amputation** |
| --- | --- | --- | --- | --- | --- | --- | --- | --- |
| Donati 2022 | Italy | 79 | 54.2 ± 16.7 | 52 M / 27 F | N/A | N/A | AK (36), BK (34), hemipelvectomy (9) | 5.1 ± 2.4 yr |
| Key findings: While lower levels of amputation and younger age predict higher prosthetic use, chronic pain syndromes (PLP, PLS, RLP) were not significantly associated with decreased prosthesis use in this sample. | | | | | | | | |
| Ernstsson 2022 | Sweden | 700 | 72.0 ± 13.5 | 474 M / 226 F | N/A | N/A | AK (98), TK (49), BK (519) | 6 mo follow-up |
| Key findings: EQ-5D-5L is superior than EQ-5D-3L in terms of validity, sensitivity, and informativity, especially in mobility and pain domains. Recommended for future outcome tracking and evaluations. | | | | | | | | |
| Kannenberg 2022 | United States | 46 | range: 40–59 | 46 M | 181 ± 7 | 98.7 ± 15.4 | BK | 16.2 ± 11.3 yr |
| Key findings: Powered Prosthetic Ankle-Foot improved pain and mobility for a subset of users, especially those with prior high pain levels. Acceptance was higher with the newer Empower model. Individual factors (e.g., motor learning) may influence benefit. | | | | | | | | |
| Lu 2022 | United Kingdom | 44 (8 nerve insertion (NI), 36 control) | NI: 45.4 ± 20.9, Control: 48.7 ± 14.4 | NI: 5 M / 3 F Control: 23 M / 13 F | N/A | N/A | NI: AK (2), TK (1), BK (5) Control: AK(14), TK (2), BK (20) | NI: mostly 1–3 yr, Control: mostly 2–4 yr |
| Key findings: Nerve insertion into adjacent muscle at amputation significantly reduces PLP and RLP and improves function. | | | | | | | | |
| Kar 2023 | Turkey | 30 (quantitative), 20 (qualitative) | 47.2 ± 16.1 | 24 M / 6 F | N/A | N/A | BK/ankle (11), knee/AK (19) | 12–24 mo |
| Key findings: QoL was below average; body image disturbance was above average. Emotional burden and functional limitations post-amputation remain substantial. | | | | | | | | |
| Seth 2023 | United States | 83 | 48.3 ± 13.4 | 46 M / 37 F | 171.4 ± 11.0 | 87.4 ± 20.2 | AK (26), BK (57) | AK: Median 7.3 yr, BK: Median 5.6 yr |
| Key findings: Multisite pain, reduced balance-confidence, and poor functional mobility are modifiable risk factors for recurrent falls in adults with LLA. Findings support using tools like PLUS-M and ABC to identify at-risk individuals and guide interventions. | | | | | | | | |

Supplementary Table 1. Detailed Characteristics of Included Studies and Participants

| **Author(s)/year** | **Country** | **Sample Size** | **Age (Mean ± SD, yr; range)** | **Sex (M/F)** | **Height (cm)** | **Weight (kg)** | **Amputation Level** | **Time since amputation** |
| --- | --- | --- | --- | --- | --- | --- | --- | --- |
| Wijekoon 2023 | Sri Lanka | 85 | 46.3 ± 6.0 | 85 M | N/A | N/A | AK (7), BK (78) | 21.7 ± 5.9 yr |
| Key findings: Veterans with LLA experience higher rates of pain and physical comorbidities and worse functional outcomes than able-bodied peers, even 10+ years post-amputation. | | | | | | | | |
| Berger 2024 | Denmark | 60 | 73.7 ± 12.1 | 35 M / 25 F | N/A | N/A | AK (36), BK (17), Bilateral (7) | ≥ 1 d |
| Key findings: Fatigue, fear of mobilization, and residual limb pain significantly limit basic mobility and completion of physiotherapy early after dysvascular lower limb amputation. These factors should be targeted in acute rehabilitation settings to enhance compliance and recovery. | | | | | | | | |
| Butowicz 2024 | United States | 21 (9 with LBP, 12 without LBP) | Total: 33.5 ± 7.6, LBP: 34 ± 9, without LBP: 33 ± 6 | N/A | Total: 180 ± 6, LBP: 181 ± 8, without LBP: 179 ± 4 | Total: 88.4 ± 14.4, LBP: 89.0 ± 17.3, without LBP: 87.9 ± 10.8 | BK | Total: 7.0 ± 4.1 yr, LBP: 6.7 ± 3.6 yr, without LBP: 7.4 ± 4.7 yr |
| Key findings: BK amputees with chronic low back pain may adopt gait strategies that minimize spinal shear forces, particularly at higher walking speeds. This alteration in mediolateral spinal loads and associated muscle activation patterns may contribute to persistent chronic low back pain. Further research on pain chronicity progression and spinal load accumulation is recommended. | | | | | | | | |
| Callskan 2024 | Turkey | 29 (15 experimental, 14 control) | Experimental: 31.2 ± 10.0, Control: 35.6 ± 10.7 | 29 M | N/A | N/A | AK | Mean 6.0 yr |
| Key findings: Kinesiology taping to the thoracolumbar fascia improved standing balance and reduced LBP in transfemoral amputees. | | | | | | | | |
| Gaffney 2024 | United States | 14 | 50.2 ± 12.0 | 7 M / 7 F | N/A | N/A | AK | 12.9 ± 9.8 yr |
| Key findings: Bone-anchored limbs may reduce LBP by decreasing compensatory trunk-pelvis movement. Improvements in ROM and CRP suggest better lumbopelvic control. Bone-anchored limbs could lead to long-term biomechanical and clinical benefits. | | | | | | | | |

Supplementary Table 1. Detailed Characteristics of Included Studies and Participants

| **Author(s)/year** | **Country** | **Sample Size** | **Age (Mean ± SD, yr; range)** | **Sex (M/F)** | **Height (cm)** | **Weight (kg)** | **Amputation Level** | **Time since amputation** |
| --- | --- | --- | --- | --- | --- | --- | --- | --- |
| Hotta 2024 | Brazil | 45 (15 amputees,  15 SCI, 15 controls) | Amputees: 35.0 ± 8.7, SCI: 31.4 ± 7.8, Control: 22.7 ± 2.5 | Amputees: 9 M / 6 F | 165 ± 7 | 73.3 ± 12.7 | N/A | N/A |
| Key findings: Orthostatic support improved function and reduced pain in amputees and SCI patients over 10 days without adverse effects. | | | | | | | | |
| Pousett 2024 | Canada | 104 (30 initial rehabilitation,  74 socket replacement) | Rehab: 65.7 ± 12.9 Replacement: 48.5 ± 16.8 | N/A | N/A | N/A | AK, BK, Bilateral BK, Rotationplasty | Rehab: 0.4 ± 1.0 (AK), 0.5 ± 1.0 yr (BK),  Replacement: 22.2 ± 14.2 (AK), 9.1 ± 12.0 yr (BK) |
| Key findings: PLUS-M is effective outcome measure across all treatment phases. 2minute walk test is more suitable for rehab phase; Minimal detectable change values guide interpretation of true clinical change in practice. | | | | | | | | |
| Schnetz 2024 | Germany | 98 (52 with arthrodesis, 46 with AK) | Arthrodesis: 74.0 ± 12.0, AK: 71.0 ± 12.7 | AK: 16 M / 30 F | N/A | N/A | AK | 77.0 ± 30.9 mo |
| Key findings: AK had significantly higher rates of complications (59% vs. 23%), mortality (28.3% vs. 3.8%), and worse functional outcomes and mobility than knee arthrodesis. Prosthesis fitting and walking ability are crucial to survival after AK. | | | | | | | | |
| Demofonti 2025 | Italy | 13 | 61.5 ± 11.1 | 8 M / 5 F | N/A | N/A | BK(6), AK(7) | 18.5 ± 21.5 mo |
| Key findings: Transcutaneous electrical nerve stimulation enhanced prosthesis embodiment, enabling greater load bearing and more physiological gait patterns | | | | | | | | |
| Fournier-farley 2025 | Canada | 22 | Median 62.5 | 16M / 6F | 170 | 85.6 | BK | 2.5 yr |
| Key findings: Residual limb neuropathic pain intensity was linked to functional and clinical factors, but not to ultrasound-derived morphological measures of neuroma. | | | | | | | | |

SCI: Spinal cord injury

Supplementary Table 1. Detailed Characteristics of Included Studies and Participants

| **Author(s)/year** | **Country** | **Sample Size** | **Age (Mean ± SD, yr; range)** | **Sex (M/F)** | **Height (cm)** | **Weight (kg)** | **Amputation Level** | **Time since amputation** |
| --- | --- | --- | --- | --- | --- | --- | --- | --- |
| Parr 2025 | United States | 58 (28 with LBP, 30 without LBP) | LBP: 38.3 ± 10.0 No LBP: 36.6 ± 12.2 | LBP: 22 M / 8 F; No LBP: 19 M / 9 F | LBP: 176.6 ± 8.5; No LBP: 176.0 ± 7.1 | LBP: 91.8 ± 19.5; No LBP: 86.2 ± 19.8 | BK | LBP: 8.9 ± 8.1; No LBP: 9.0 ± 14.5 |
| Key findings: Individuals with low back pain showed greater medial–lateral movement variability, particularly in acceleration and jerk measures during sit-to-stand and stand-to-sit. | | | | | | | | |
| Penasso 2025 | Austria | 18 | 53.5 ± 15.0 | 9 M / 9 F | 184.5 ± 9.0 | 68.4 ± 13.9 | BK | 18.3 ± 19.2 mo |
| Key findings: Vibrotactile feedback showed a small positive effect on balance and gait in slower-walking participants, with the strongest impact on four-square-step-test performance, while stance time remained unchanged. | | | | | | | | |
| Rierola-Fochs 2025 | Spain | 20 (10 experimental, 10 control) | Experimental: 55.2 ± 11.2, Control: 66.3 ± 7.6 | 19 M / 1 F | N/A | N/A | LLA (16) | Experimental: 10 mo (IQR: 2–40), Control: 12 mo (IQR: 1–220) |
| Key findings: The home-based Graded Motor Imagery protocol is feasible for home-based implementation in individuals with PLP. Participants adhered well (89.32%), found mobile tech usable, and showed meaningful pain reductions. Clinical trials are warranted. | | | | | | | | |
| Shaw 2025 | United States | 54 | BK: 53.9 ± 12.3, AK: 51 ± 11.3 | BK: 13 M / 9 F; AK: 17 M / 15 F; | N/A | N/A | BK (22),  AK (32) | BK: 8.7 ± 10.2 yr, AK: 14.6 ± 13.4 yr |
| Key findings: At 1 year, patients showed significant improvements in pain, global health, and quality of life, including reduced residual limb pain during walking, across both transfemoral and transtibial groups. | | | | | | | | |
