# Supplementary material for: Assessment of pain and functional outcomes after lower limb amputation: a scoping review
Source: BMJ Open. 2026 Mar 10;16(3):e110319. doi: 10.1136/bmjopen-2025-110319 (PMC12983873; doi:10.1136/bmjopen-2025-110319)
Supplement: online supplemental table 2 [file bmjopen-16-3-s003.docx]

Supplementary Table 2. Pain & Functional Assessments and Outcomes

| Author | Pain type | Pain  Assessments | Pain Outcomes | Functional Assessments | Functional Outcomes | Study Summary |
| --- | --- | --- | --- | --- | --- | --- |
| Persson and Liedberg, 1982 | RLP, PLP | Self-reported /observed | RLP (27.1%), No RLP (72.9%), PLP (21%), No PLP (79%) | Spring scale mounted on an adjustable table | MEW (% body weight): RLP (10.9 ± 6.8), No RLP (19.5 ± 14.1); PLP (17.3 ± 18.9), No PLP (16.5 ± 11.4) | RLP associated with lower MEW (p = 0.006); PLP not significant (p = 0.83). |
| Helm et al., 1986 | RLP, PLP | Self-reported | RLP (44%), PLP (71%) | Prosthesis use/mobility and living support needs | Function decreased with intense (β = 0.96) and mild pain (β = 0.75); no significant effect on dependence (β = 0.22, 0.09). | Pain reduced physical function (β = 0.75–0.96, p < 0.05), but did not significantly affect social dependence (β = 0.09–0.22, p > 0.05). |
| Kelly et al., 1998 | RLP | VAS  (Scale: 0–10) | Wide variation: 0–78.8% of maximum; pain >33% correlated with slower walking. | VA-Rancho Stride Analyzer  (Gait parameters: velocity, cadence, stride length, gait cycle, single/double-limb support) | No consistent gait changes when pain <33.5%, but all subjects slowed down when pain >33.5%; other parameters (stride length, cadence, SLS) showed no consistent correlation | Significant correlation between pain intensity and change in gait velocity (p = 0.046); high pain (>33%) associated with slowing, others showed variable responses. |
| Hoogendoorn and Van Der Werken, 2001 | RLP | SF-36 Bodily Pain scale, Customed questionnaire item on pain frequency | 50% of amputees reported no pain, 39% occasional pain, 11% continuous pain. | NHP (mobility score), SF-36 Physical Functioning,  Customed questionnaire on walking distance, work, disability | 28% walked <100 m, 44% walked 100 m–5 km, and 28% walked >5 km; 61% reported problems with profession, and 50% felt disabled. | 72% reported walking limitations (<5 km); SF-36 PF scores ranged 53–58. 28% reported health interfered with work. |
| Jones et al., 2001 | RLP | VAS (Scale: 0–100) | VAS: 49.2  → 21.1 (p < 0.000) | SWB, Walking Velocity (m/s over 10 m) | Velocity: 0.28 → 0.51 m/s; SWB: 61.3% → 81.5% BW. | Pain reduced SWB (β = –0.48), which improved velocity (β = 0.42). |

MEW: Maximal end-weight bearing; NHP: Nottingham Health Profile; SWB: Static Weight Bearing

Supplementary Table 2. Pain & Functional Assessments and Outcomes (Continued)

| Author | Pain type | Pain  Assessments | Pain Outcomes | Functional Assessments | Functional Outcomes | Study Summary |
| --- | --- | --- | --- | --- | --- | --- |
| Van Der Schans et al., 2002 | PLP, RLP | GQPLA; RAND-36 DLV pain domain | PLP (80%), RLP (68%) | Walking distance (categorized: >1 km, 500–1000 m, 100–500 m, <100 m); RAND-36 DLV | PLP group walked 100–500 m; without PLP walked 500–1000 m. PLP group scored 60.5 on RAND-36; without PLP scored 67.5. | PLP associated with ~14-point reduction in QoL (p < 0.05); RLP linked to 5.1–15 point lower QoL. Amputees walking <500 m scored 10.2–31.8 points lower than those walking >500 m (p < 0.001). |
| Chou et al., 2003 | RLP | NRS  (Scale: 0–10) | Pain increased with walking speed (slow 0.66 → normal 1.60 → fast 1.53) | 3D motion analysis (OrthTrak) for kinematics/kinetics; Pedar system for interface pressure | Significant increases in hip flexion (p = 0.013), hip extension (p = 0.020), knee extension (p = 0.006), and ankle dorsiflexion (p = 0.017) with speed. | Speed increased ROM and joint loading, but not pressure or pain. Multi-axis foot moderately absorbed shock; pressure aligned with pain. |
| Rudy et al., 2003 | LBP | West Haven–Yale MPI, MPQ-SF, NRS (Scale: 0-10) | Pain frequency: daily (n = 14), 4–6x/week (n = 2), 2–3x/week (n = 3 | Force plate for sway measures (AP and lateral sway), Performance outcome: number of lifts/reps, lift speed; | Chronic pain group had fewer lifts (0.84 vs 1.16), fewer push–pull repetitions (0.85 vs 1.15), and a lower late–early lifting speed ratio (0.64 vs 0.68); sway measures showed no notable differences | Chronic pain impaired performance (p < 0.05) but did not affect movement mechanics (p > 0.2). Pain catastrophizing was negatively associated with performance. |
| Mackenzie et al., 2004 | n.a. | VAS  (Scale: 0–100) | Mean VAS pain: BK 24.7 ± 23.7; TK 14.4; AK 27.0 | SIP, FIM | SIP scores (physical function): BK 9.8 ± 10.6, TK 13.9 ± 9.0, AK 9.0 ± 7.2; FIM (Walking): BK 98.8%, TK 87.5%, AK 96.3% | n.a. |

GQPLA: Groningen Questionnaire Problems Leg Amputation; RAND-36 DLV: Dutch version of SF-36; QoL: (Health related) Quality of life; MPI: Multi-dimensional Pain Inventory; MPQ: McGill Pain Questionnaire; AP: Anterio-posterior SIP: Sickness Impact Profile; FIM: Functional Independence Measure

Supplementary Table 2. Pain & Functional Assessments and Outcomes (Continued)

| Author | Pain type | Pain  Assessments | Pain Outcomes | Functional Assessments | Functional Outcomes | Study Summary |
| --- | --- | --- | --- | --- | --- | --- |
| Williams et al., 2004 | PLP, RLP | BPI | MSPSS associated with lower pain interference (p < 0.05) | CHART-M (Mobility),  CHART-OF (Occupational Function) | Higher MSPSS predicted better mobility and occupational functioning at 6 months (p < 0.05) | 1 month post-amputation: Higher social support decreased pain interference, increased mobility;  6 months: No significant link between social support and pain or mobility |
| Berge et al., 2005 | RLP | CPG (Scale: 0-10) | Mean CPG pain: 3.0 ± 3.2 | Gait parameters (accelerometer), Vertical ground reaction force, prosthetic side knee angle, Steps per week. | Walking speed: 1.25 ± 0.19 m/s, Step length: 0.70 ± 0.07 m, Loading rate: 8.99 ± 2.75 BW/s, Deceleration peak: 1.11 ± 0.10 BW, Knee angle: 2.71 ± 5.71°, Steps/week: 21,241 ± 11,902. | n.a. |
| Friel et al., 2005 | LBP | VAS  (Scale: 0–10), revised-ODI | VAS score: range 0–6.9 (Mild LBP 80%, Moderate LBP 5%, Severe LBP 15%); Revised-ODI significantly higher in LBP group (e.g., mean 23.8% vs 3.4%; p < .001). | Iliopsoas length; Hamstring length; Abdominal strength;  Back extensor strength;  Back endurance | Iliopsoas length: no-pain –12.4, pain –1.8;  Back extensor strength: no-pain 3.4, pain 2.3;  Hamstring length: no-pain –29.5, pain –22.1;  Abdominal strength: no-pain 4.1, pain 3.8;  Back endurance: no-pain 30.1, pain 13.5 | Back extensor endurance significantly reduced in LBP group (mean 13.5s vs. 30.1s; p = 0.068). Iliopsoas length and back extensor strength were also lower in LBP group. |

BPI: Brief Pain Inventory; MSPSS: Multidimensional Scale of Perceived Social Support; CHART: Craig Handicap Assessment and Reporting Technique; CPG: Chronic Pain Grade; ODI: Oswestry Disability Index

Supplementary Table 2. Pain & Functional Assessments and Outcomes (Continued)

| Author | Pain type | Pain  Assessments | Pain Outcomes | Functional Assessments | Functional Outcomes | Study Summary |
| --- | --- | --- | --- | --- | --- | --- |
| Kulkarni et al., 2005 | LBP | VAS  (Scale: 0–10) | 63% reported back pain; 27% severe (VAS 7–8), 11% extreme (VAS 9–10). Strong co-occurrence with phantom (62%) and stump pain (57%). 81% of AK had LBP vs. 62% of BK (p < 0.05). | Gait lab analysis (GRFs, walking speed), Standing stability analysis (centre of pressure displacement), Lumbar MRI, Physical examination | Walking speed: Pain-free group (1.26 ± 0.18 m/s), Pain group (1.06 ± 0.25 m/s); vertical impact force peak: Pain-free group (1.218 ± 0.156), Pain group (1.084 ± 0.123 m/s); | Pain-free amputees walked faster (p < .05); had greater GRF during impact. No significant asymmetry in gait patterns. Pain group had more postural sway in standing, especially with vision occluded (216% greater contact area). |
| Norvell et al., 2005 | RLP, ILP | CPG  (Scale: 0–10) | Knee pain prevalence: AK 50%, BK 36.4%, Nonamputees 20.2%. Pain was more prevalent in the intact limb, especially in AK amputees. | Self-report CPG items (pain intensity, disability days, interference with daily life) | Amputees: CPG Grade I (28%), Grade II (40%), Grade III (16%), Grade IV (16%); Daily activities interference (2.8 ± 2.9)  Nonamputees: CPG Grade I (19%), Grade II (38%), Grade III (31%), Grade IV (12%); Daily activities interference (3.8 ± 3.1) | Pain more likely to interfere with recreation/social activities in nonamputees. Survey data suggests higher functional burden in AK; knee of amputated limb had 5 times lower pain prevalence than intact limb. |
| Graham et al., 2006 | RLP | Self-reported | 60% reported significant stump pain | SIGAM mobility grade, Prosthesis usage patterns (duration) | SIGAM grades mostly between C and D. Prosthesis use averaged 11.2 hours/day. | Significant stump pain was significantly associated with reduced prosthesis use and lower SIGAM scores (p < 0.01). Stump pain was significantly linked to poorer mobility and psychological well-being. |

SIGAM: Special Interest Group for Amputee Medicine

Supplementary Table 2. Pain & Functional Assessments and Outcomes (Continued)

| Author | Pain type | Pain  Assessments | Pain Outcomes | Functional Assessments | Functional Outcomes | Study Summary |
| --- | --- | --- | --- | --- | --- | --- |
| Aksnes et al., 2008 | muscular pain | Self-reported /observed, SF-36 (Bodily pain domain) | Amputees reported significantly more bodily pain compared to limb-sparing group (SF-36 bodily pain scores significantly lower for amputees, p<0.01). | MSTS score, TESS, SF-36 | Amputees had significantly lower MSTS scores (median 60%) vs limb-sparing (median 80%, p<0.001). No significant difference in TESS between groups (90%). Poorer MSTS scores (<50%) strongly associated with reduced SF-36 physical and emotional functioning | Chronic muscular pain or stiffness was more physically inactive (chi-squared Fisher’s exact test, p = 0.004) than those without chronic pain. Muscular pain was a strong independent predictor of functional impairment, increasing the risk by over 4.7 times (p = 0.001). |
| Hagberg et al., 2008 | PLP, RLP | SF-36 Bodily Pain Scale, Q-TFA Problem Score (including RLP, PLP, skin issues) | Significant improvement in SF-36 Bodily Pain score (SF-36 BP score: 53 → 72, p = 0.029); Q-TFA Problem score improved by 21 points (p = 0.002). | SF-36 (generic Health-Related QoL), Q-TFA (prosthetic use, mobility, problems, global health) | Q-TFA Mobility score improved by 17 points (p = 0.001); increased daily prosthetic use in 16/17 participants; 12 used prostheses >13 hours/day. Walking habit subscore improved from 39 to 57 (p = 0.013). | Osseointegrated prostheses significantly improved general and amputation-specific quality of life in transfemoral amputees over a 2-year follow-up. Improvements were seen in pain, mobility, prosthesis use, and global health perception. |
| Smith et al., 2008 | LBP, RLP | VAS  (Scale: 0–10) | Back pain in 47.7% (mean intensity: 5.3 ± 2.1); RLP in 56.1% (mean: 5.7 ± 2.3); pain mostly intermittent and moderate in intensity. | SIGAM mobility grading system | SIGAM C or better (minimum indoor ambulation ability). | Pain interference with ADLs: Back pain = 3.5, RLP = 2.9; Interference with social life: Back pain = 3.8, RLP = 2.8; Interference with work: Back pain = 3.4, RLP = 3.9 |

MSTS: Musculoskeletal Tumor Society, TESS: Toronto Extremity Salvage Score; Q-TFA: Questionnaire for persons with a Transfemoral Amputation; ADL: Activities of Daily Living

Supplementary Table 2. Pain & Functional Assessments and Outcomes (Continued)

| Author | Pain type | Pain  Assessments | Pain Outcomes | Functional Assessments | Functional Outcomes | Study Summary |
| --- | --- | --- | --- | --- | --- | --- |
| Morgenroth et al., 2009 | LBP | CPG  (Scale: 0–10),  Self-report | moderate pain (mean current = 4.3 ± 2.0; mean past 3 months = 4.8 ± 1.8); low interference with function (2.2/10 on CPG scale) | Vicon motion analysis system (with Plug-in Gait model) | Static LLD (no LBP 8.4, LBP 8.3mm), Dynamic SLS LLD (no LBP 28.4, LBP 18.4mm), Prosthetic DLS LLD (no LBP 11.0, LBP 15.1mm), Intact DLS LLD (no LBP 15.1, LBP 15.8mm). | No significant relationship found between either static (*p* = 1.0) or dynamic (*p* = 0.3) LLD and LBP in AK. Challenges common clinical assumption that LLD contributes to LBP. Suggests LBP in AK is likely multifactorial. |
| Tekin et al., 2009 | RLP | VAS  (Scale: 0–10) | VAS significantly lower in amputees (1.9 ± 0.74) vs salvage group (3.33 ± 1.12) | FAS, 6MWT, 10MWT, EEI | No significant differences between groups in 6MWT, 10MWT, or EEI at all speeds/inclines. All participants were FAS Grade 5 (independent ambulation). | Amputees reported better general health and vitality (SF-36), and less pain than salvage patients (p = 0.004). Functional performance was similar across both groups (p > 0.05). |
| Morgenroth et al., 2010 | LBP | CPG  (Scale: 0–10) | Avg. current pain: 4.3, Avg. 3-month pain: 4.8 | Vicon 3D motion analysis; lumbar spine ROM in sagittal, coronal, and transverse planes derived from thoracic and pelvic markers | Transverse ROM: LBP group 15.4°, No pain group 11.1°. Coronal ROM: LBP group 6.6°, No pain group 8,1°. Walking speed: LBP group 1.09m/s, No pain group 1.02m/s. | Greater transverse plane lumbar spine motion during gait is associated with presence of LBP in AK (p = 0.029). No differences in coronal ROM (p = 0.2). No significant difference in walking speed or prosthesis use (p = 0.6). |

LLD: Leg-length discrepancy; SLS: Single Limb Support; DLS: Double Limb Support; FAS: Functional Ambulation Scale; 10MWT: 10-Meter Walk Test; EEI: Energy Expenditure Index

Supplementary Table 2. Pain & Functional Assessments and Outcomes (Continued)

| Author | Pain type | Pain  Assessments | Pain Outcomes | Functional Assessments | Functional Outcomes | Study Summary |
| --- | --- | --- | --- | --- | --- | --- |
| Vincent et al., 2010 | Neuropathic pain | Modified BPI | Question 5: >5/10, Question 9a: 7/10 | LCI, TUG, BBT / AMP, LSA, LIFE-H, HAP | LCI: >21/42, TUG: >14sec, BBT: <45, AMP: <25/47, LIFE-H: <7/9, LSA, HAP: <standard values for the age | Negative modulators (pain) interact with potential mobility in such a way that low effective mobility is observed. LLAs show mismatches between lab-based capabilities and real-world mobility due to social and health barriers. |
| Couture et al., 2011 | PLP, RLP | NRS (Scale: 0–10) | Stump pain: 3.95 ± 2.85, Phantom pain: 4,35 ± 3.22 | LCI; SMAF for functional independence | LCI: 35.2 ± 7.6, SMAF: 14.7 ± 9.5 | Positive appraisal of amputation was associated with less pain (p=0.06), greater body image satisfaction, and functional independence (p = 0.05). |
| Devan et al., 2012 | LBP | NRS (Scale: 0–10) | LBP prevalence = 64.1%. 14% had severe LBP (NRS ≥ 7). 38.7% had chronic LBP (>3 years without pain-free month). | PASIPD – measuring MET hours/day | Low (≤13.6 MET h/d, n=48), Medium (13.6–32.5 MET h/d, n=48), and High (≥32.5 MET h/d, n=49) activity levels. | No direct link between overall physical activity levels and presence of LBP. However, physical activity is reduced in those reporting activity restriction due to LBP (p = 0.02). |
| Fortington et al., 2013 | PLP, RLP | RAND-36 Pain Subscale (Scale: 0–100) | Baseline (26.2 ± 3.2), 6 months (47.8 ± 5.2), 18 months (42.8 ± 5.3). | Health-related QoL + self-reported walking distance | Physical function: Baseline (12.0 ± 1.7), 6 months (21.4 ± 3.8), 18 months (22.6 ± 4.2). Walking declined in 32% at 6 months but improved or stabilized by 18 months | Pain and physical function both improved significantly from baseline to 6 months (*p* < 0.001) and were maintained at 18 months (*p* < 0.001). |

TUG: Timed Up and Go; BBT: Berg Balance Test; AMP: Amputee Mobility Predictor; LSA: Life Space Assessment; LIFE-H: Assessment of Life Habits; HAP: Human Activity Profile; LCI: Locomotor Capabilities Index; SMAF: Functional Autonomy Measurement System; PASIPD: Physical Activity Scale for Individuals with Physical Disabilities; MET: Metabolic equivalent

Supplementary Table 2. Pain & Functional Assessments and Outcomes (Continued)

| Author | Pain type | Pain  Assessments | Pain Outcomes | Functional Assessments | Functional Outcomes | Study Summary |
| --- | --- | --- | --- | --- | --- | --- |
| Hagberg et al., 2014 | PLP, LBP, ILP | Q-TFA | Baseline: 43 ± 19.1, 2-year follow-up: 16 ± 11.3 | SF-36 PF, SF-36 PCS | PF: Baseline 35.7 ± 21.3 → 2-year follow-up 60.0 ± 21.4 PCS: Baseline 32.1 ± 9.1 → 2-year follow-up 40.5 ± 9.8 | Pain significantly decreased (43 → 16, *p* < 0.0001), while physical function improved markedly (35.7 → 60.0, *p* < 0.0001) over two years, suggesting concurrent improvements in pain and functional outcomes. |
| Roth et al., 2014 | RLP | NRS (Scale: 0–10) | SNF: 3.3 ± 2.7, IRF: 2.9 ± 2.5, Home health: 2.9 ± 2.7 | PEQ-Utility | SNF: 1.7 ± 1.4, IRF: 2.4 ± 1.1, Home health: 2.5 ± 1.2 | Pain was low and similar across settings (p=0.80), but prosthetic use was higher in IRF and home health vs. SNF (p=0.04), showing better function. |
| Russell Esposito and Wilken, 2014 | LBP | PEQ – LBP Frequency item | LBP group (n = 9) described frequency ≥ 1×/week | 3D motion capture system; CRP and CRP variability calculated in sagittal, frontal, and transverse planes | Compared to the noPain group, LBP group showed less trunk lean, greater pelvic motion, reduced trunk rotation, and more in-phase frontal coordination at higher speeds. | LBP was associated with less trunk lean (p < 0.001), greater pelvic motion (p < 0.001), reduced trunk rotation (p = 0.003), and more in-phase frontal coordination at higher speeds (p = 0.014). |
| Segal et al., 2014 | RLP | Revised CPG | Avg. RLP: 2.9 ± 1.3 (rigid adapter), 2.3 ± 1.7 (torsion adapter). | 6MWT, SAM | 6MWT: 463 ± 76 vs. 464 ± 81 m. Steps/day: total 6269 vs. 6728; low-intensity 2197 vs. 2528; medium 3034 vs. 3470; high 1038 vs. 730 (rigid vs. torsion; all ±SD). | Torsion adapters reduced pain interference (*p* = 0.026) and increased low- and medium-intensity activity (*p* = 0.019, *p* = 0.024) but had no effect on average pain (*p* = 0.23) or 6MWT performance. |

PF: Physical Functioning; PCS: Physiological Component Summary; SNF: Skilled Nursing Facility; IRF: Inpatient Rehabilitation Facility; PEQ: Prosthesis Evaluation Questionnaire; CRP: Continuous Relative Phase; SAM: Step Activity Monitor

Supplementary Table 2. Pain & Functional Assessments and Outcomes (Continued)

| Author | Pain type | Pain  Assessments | Pain Outcomes | Functional Assessments | Functional Outcomes | Study Summary |
| --- | --- | --- | --- | --- | --- | --- |
| Ladlow et al., 2015 | n.a. | DMRC Pain Status Score | No pain: 26%, controlled pain: 72%, uncontrolled pain: 2%. | 6MWT, AMP-Pro, SIGAM Mobility Grade, DMRC Mobility and ADL Score | Mean 6MWT distance = 489 ± 117 m; AMP-Pro score = 43.5 ± 5; 91% achieved SIGAM F (independent walking). | Despite high injury severity (NISS = 40), 95% of military amputees were independent in ADLs, 85% could walk or run independently, and 98% reported controlled pain; mental health outcomes showed no significant group differences (*p* > 0.05). |
| Soin et al., 2015 | PLP, RLP | NRS (Scale: 0–10), BPI | NRS: Baseline 6.6 ± 0.4, 12 months 3.3 ± 0.5; BPI: Baseline: 5.7 ± 0.4, 12 months 2.5 ± 0.5 | BPI interference subscale (ADL, walking, work, etc.) | Pain interference decreased from ~7.8 to ~3.0 for walking ability, and from ~5.8 to ~2.8 for general activity after treatment. | Average pain scores decreased by over 50% from baseline, and this was accompanied by significant reductions in pain interference —walking ability (*p* < 0.01), general activity (*p* < 0.01). |
| Anaforoğlu et al., 2016 | LBP | VAS (Scale: 0–100) | Experimental group: Pre 5.71 ± 2.56 to Post 2.86 ± 2.41; Control group:  Pre 5.93 ± 2.67 to Post 4.71 ± 2.81. | ODI, Spinal flexibility (Sit-and-reach test, spinal extension/lateral flexion/rotation flexibility tests) | Experimental group: ODI 14.35 to 4.65; Trunk flexion 15.67 to 22.30; Right lateral flexion 17.52 to 20.68; Right rotation 13.57 to 16.19. Control group: ODI 16.45 to 9.85; Trunk flexion 15.10 to 16.10; Right lateral flexion right 17.10 to 17.95; Right trunk rotation. | Pain was significantly lower in the experimental group (*p* < 0.001), with also significant improvements in trunk flexion (*p* = 0.009), right lateral flexion (*p* = 0.046), right rotation (*p* = 0.010), and ODI (*p* = 0.001). |

DMRC: Defence Medical Rehabilitation Centre; AMP-Pro: Amputee Mobility Predictor with Prosthesis;

Supplementary Table 2. Pain & Functional Assessments and Outcomes (Continued)

| Author | Pain type | Pain  Assessments | Pain Outcomes | Functional Assessments | Functional Outcomes | Study Summary |
| --- | --- | --- | --- | --- | --- | --- |
| Fatone et al., 2016 | LBP | VAS (Scale: 0–100) | LBP group pain intensity mean: **3.1 ± 1.3**, No-LBP group pain intensity mean: 0.7 ± 0.7 | 3D motion analysis system (120Hz); kinematic analysis of pelvis, lumbar, and thoracic spine using reflective markers and software (Visual3D) | Sagittal plane lumbar ROM: LBP 5.6° ± 2.2°, No-LBP 7.3° ± 2.9°; Pelvic tilt ROM: LBP 11.5° ± 4.5°, No-LBP 12.0° ± 4.3°; No significant differences in frontal and transverse planes between groups. | No statistically significant correlations were found between pain intensity and spinal or pelvic kinematic variables (all *p* > 0.05). Lumbar spine sagittal ROM tended to be lower in the LBP group, but this difference was not statistically significant (p = 0.08). |
| Ladlow et al., 2016 | RLP | DMRC-developed pain status score | Controlled or no pain (97%); uncontrolled pain was higher in limb salvage (14%) vs. unilateral amputees (3%). | 6MWT, DMRC Mobility and ADL Score | 6MWT: Unilateral amputees = 564 ± 92 m; limb salvage = 483 ± 108 m (p < 0.05); delayed BK amputees = 595 m; bilateral amputees = 409 m. | Better functional outcomes (greater walking distance and independent mobility) in amputation groups correlated with better pain control (p-values for group differences in 6MWT < 0.05). |
| MacKenzie et al., 2016 | RLP, LBP | Self-reported/observed | RLP (24%), back pain (12%), and skin problems (6%) were common MWT barriers. | MWT, TUG, 2MWT, L Test of functional mobility | MWT completed by 78% with gait speed 0.37 m/s (limited) vs. 0.59 m/s (max); 54% stopped early due to fatigue (53%), pain, or skin issues. | Pain caused 24% of incomplete tests. Length of stay (p = 0.007), walking aid type, and amputation level (p < 0.001) significantly affected MWT completion. Fatigue and pain limited functional mobility. |
| Devan et al., 2017 | LBP | NRS (Scale: 0–10) | LBP prevalence was 63%. (>4 weeks) | Functional activity questions based on ODI (e.g., pain while sitting, standing, sit-to-stand, climbing stairs, etc.) | Not directly assessed with objective measures but inferred from functional activity questions. Sit-to-stand difficulty was significantly associated with higher LBP intensity. | LBP intensity (NRS) was linked to residual limb issues (β=0.21, p=.01), LBP during sit-to-stand (β=0.22, p=.03), and lower in employed individuals (β=-0.18, p=.03). |

MWT: Maximum walk test; 2MWT: 2-Minute Walk Test

Supplementary Table 2. Pain & Functional Assessments and Outcomes (Continued)

| Author | Pain type | Pain  Assessments | Pain Outcomes | Functional Assessments | Functional Outcomes | Study Summary |
| --- | --- | --- | --- | --- | --- | --- |
| George et al., 2017 | PLP | Self-reported/observed | 64% experienced phantom pain, 32% had persistent phantom pain (>3 months) | Ambulatory status questionnaire, VR-12 | At minimum 1-year follow-up (n=43): Non-ambulators 47%, Home ambulators 28%, Community ambulators 26%; Ambulatory status after AKA was similar to preoperative status (p = 0.961) | Persistent phantom pain was significantly associated with poor ambulation (Odds Ratio 0.09, 95% CI 0.02–0.60, p = 0.012) |
| Mahon et al., 2017 | LBP | Overuse-related kinematic variables were used to infer risk of low back pain | Overuse-related indicators (e.g., trunk flexion, vGRF loading) suggest elevated low back and knee joint loading. | motion capture (Vicon), 6 force plates, CHAMP, 6MWT, PEQ, oxygen cost via metabolic system | 6MWT: median 509 m (range 360–704); CHAMP median: 20/40. Increased trunk velocity and step width variability. Higher oxygen cost (0.18 mL/kg/m vs 0.15 in controls). Larger vGRF on intact limb. Reduced stance symmetry. | As increased trunk lateral flexion is associated with larger spinal loads, over time, there is likely an increased risk of low back disorders and/or pain. |
| Atiç and Aydın, 2018 | RLP | Self-reported /observed; RLPS (Scale: 0–10). | Mean RLPS: Vascular (1.98 ± 1.76), Trauma (1.87 ± 1.78), Landmine (1.93 ± 2.01) | 10MWT (speed, sec), 6MWT (distance, meters), daily walking distance (meters/day), daily prosthesis use time (hours/day) | Vascular vs. Trauma groups: Walking speed 32.43 ± 5.01 sec vs. 23.81 ± 3.49 sec; 6MWT 121.64 ± 16.65 m vs. 266.10 ± 71.74 m; Walking distance/day 947.62 ± 228.71 m vs. 1400.00 ± 272.74 m; Walking time without pain 53.10 ± 18.51 min vs. 68.33 ± 21.05 min | Despite similar residual limb pain levels (p = 0.212 between vascular and trauma), trauma and landmine amputees showed significantly better functional outcomes than vascular group (p < 0.001 for most functional parameters). Lower pain interference reported alongside higher physical function and prosthetic satisfaction in trauma and landmine groups. |

VR-12: Veterans RAND 12-Item Health Survey; CHAMP: Comprehensive High-level Activity Mobility Predictor; RLPS: Residual Limb Pain Scale

Supplementary Table 2. Pain & Functional Assessments and Outcomes (Continued)

| Author | Pain type | Pain  Assessments | Pain Outcomes | Functional Assessments | Functional Outcomes | Study Summary |
| --- | --- | --- | --- | --- | --- | --- |
| Aydın and Çağlar Okur, 2018 | RLP | VAS (Scale: 0–10) | Resting pain: Exp. group 2.1 ± 1.2, Control group 2.7 ± 1.4; Walking pain: Exp. group 3.2 ± 1.7, Control group 4.3 ± 2.1 | Daily walking distance (metres), 10MWT (seconds), 10-step climbing up/down, 10-metre walking up/down 8% slope | Experiment vs. Control: Daily walking distance (m) 1332.14 vs. 1047.62; 10-step climbing up (sec): 20.07 vs. 25.76; 10m walking up 8% slope (sec): 28.13 vs. 35.19 | Lower walking pain was associated with better function and satisfaction (significance reflected in p-values < 0.05). |
| Esfandiari et al., 2018 | PLP, RLP, LBP | Self-reported/observed | Phantom pain 63%, Stump pain 49%, Back pain 69%, Contralateral joint pain 67% | AMP-Pro | Mean AMP Pro score: 34.7 (TK), 32.8 (AK), 31.7 (TH). | Longer stump length in AK was significantly associated with better AMP-Pro score (p = 0.014). Patients with higher-level amputations had more pain and lower function |
| Golyski and Hendershot, 2018 | LBP | VAS (Scale: 0–10) | No participants reported moderate/severe pain during testing (VAS < 4). | 3D motion capture, TAM/RAM, trunk-pelvis coordination using vector coding in sagittal, frontal, and transverse planes | Sagittal trunk-pelvis ROM rose during step turns (8.9° vs. 6.5°), frontal ROM dropped during spin turns (11.4° vs. 15.3°), and LLA showed greater trunk momentum and pelvic coordination. | Altered trunk-pelvic dynamics observed in LLA may increase risk for low back pain development. Statistical significance for altered kinematics and coordination is indicated by *p* values (p < 0.05). |
| Allami et al., 2019 | PLP, RLP, LBP, Neuropathic pain | NRS (Scale: 0-10), DN4 questionnaire (for neuropathic pain) | Stump pain 84.2%, intensity 3.2; Phantom pain 73.7%, intensity 2.3; Low back pain 78.1%, intensity 3.4; Knee pain 54.7% | Self-reported prosthesis use (hours/day and meters/day walking or standing with prosthesis) | Average prosthesis use: 7 days/week, 12.47 ± 3.84 hours/day, walking or standing ~4.22 ± 3.53 hours/day, average distance ~2826.73 ± 2679.83 meters/day. | PLP increased RLP odds (p = 0.012), RLP raised LBP odds (p = 0.002), higher LBP intensity increased RLP odds (p = 0.003), and more daily prosthesis use reduced LBP odds (p = 0.039). |

TAM: Translational angular momentum; RAM: Rotational angular momentum; DN4: Douleur Neuropathique en 4 Questions

Supplementary Table 2. Pain & Functional Assessments and Outcomes (Continued)

| Author | Pain type | Pain  Assessments | Pain Outcomes | Functional Assessments | Functional Outcomes | Study Summary |
| --- | --- | --- | --- | --- | --- | --- |
| Butowicz et al., 2019 | LBP | VAS (Scale: 0-10) | LBP defined as pain ≥3 months, ≥50% of days in past 6 months.  Pain intensity: LBP group ~1.8 ± 1.5 | Unstable sitting task with 3D motion capture, Trunk kinematics and COP metrics, Surface EMG of lumbar/thoracic erector spinae, ODI | ODI was 29.9 ± 24.5 in the LBP group and 7.4 ± 9.1 without LBP. The LBP group showed asymmetrical lumbar muscle activation, greater anterior-posterior trunk motion, and reduced dynamic stability | LBP group had higher ODI scores (p = 0.002), larger anteroposterior trunk motion (p = 0.03), and reduced trunk stability (p ≤ 0.005); COP differences were not significant despite medium to large effect sizes. |
| Çalışkan Uçkun et al., 2019 | RLP | VAS (Scale: 0-10) | Mean residuum pain scores: PIN/LOCK group 1.1 ± 2.2, Vacuum-assisted suspension system (VASS) group 1.8 ± 2.8 | Physical activity (PA) measured by IPAQ-SF, SF-36 | Total PA (MET-min/week): PIN/LOCK 1387.2 ± 2352.2, VASS 1014.1 ± 978.9, Controls 1879.3 ± 1260.2. Both prosthesis groups had lower physical activity and SF-36 scores than controls. | Physical activity level correlated significantly only with the SF-36 physical functioning subscale (r = 0.303, p = 0.031). Other factors such as age, BMI, cigarette use, and residual limb pain were not significantly correlated with total physical activity. |
| Dakhil et al., 2019 | RLP | Wong-Whaley pain scale (Scale: 0–10); PEQ | 7/8 participants reported minor to moderate pain mostly localized in proximal/distal regions; one participant reported severe pain in the knee region. | Measure static and dynamic interface pressure; during 2-min walk along 6 m path. PEQ | Highest pressure recorded: Static: 121.1 ± 31.6 kPa (proximal stump); Dynamic: 254.1 ± 61.2 kPa (popliteal area during gait loading). | Higher static peak pressures strongly associated with increased pain scores (p = 0.03). A threshold of <0.9 kPa/kg may help ensure long-term satisfaction. |

COP: Centre of Pressure; IPAQ-SF: International Physical Activity Questionnaire – Short Form

Supplementary Table 2. Pain & Functional Assessments and Outcomes (Continued)

| Author | Pain type | Pain  Assessments | Pain Outcomes | Functional Assessments | Functional Outcomes | Study Summary |
| --- | --- | --- | --- | --- | --- | --- |
| Facione et al., 2019 | LBP | VAS (Scale: 0-10), ODI | Mean max VAS in AK-LBP group: 6.5/10; ODI impact = 16.4%. AK-without LBP group had no pain. 41.7% total prevalence of LBP in the sample. | Spinal curvature and pelvic alignment measures. T9-tilt, Sagittal Vertical Axis, OD-HA, TK, Cervical Lordosis, Maximal Lumbar Lordosis, Pelvis Tilt, Sacral Slope | TK angle: LBP group 33.5°, No-LBP group 41.3°. T9-tilt angle: LBP group 11.2°, No-LBP group 7.2° | Difference in TK angle between groups: *p* = 0.051. Difference in T9-tilt angle between groups: *p* = 0.046. No significant differences in ODI or lumbar/pelvic parameters between groups. |
| Gilmore et al., 2019 | PLP, RLP | NRS (Scale: 0–10), BPI-Short Form | Pain intensity decreased by up to 50% for RLP and 56% for PLP during PNS therapy. By week 8, 58% of PNS patients had ≥50% pain reduction versus 14% placebo, with relief lasting up to 12 months. | BPI interference scale (pain impact on ADLs); Patient Global Impression of Change (PGIC) | 80% of PNS group had ≥50% reduction in pain interference at 8 weeks (p = 0.003). Significant improvements in PGIC (2.2 ± 0.9 at 8 weeks vs. 0.6 ± 1.3 in placebo). | More PNS patients achieved ≥50% pain relief than placebo at weeks 1–4 (58% vs. 14%, p=0.037) and 5–8 (67% vs. 14%, p=0.014). Pain interference dropped ≥50% in 80% of PNS patients vs. 15% placebo at week 8 (p=0.003). PGIC scores favoured PNS (p=0.007). Opioid use changes were similar between groups (p>0.999). |
| Külünkoğlu et al., 2019 | PLP | VAS (Scale: 0-100) | MT group 70.5 to 0, PE group 67.5 to 6.5. | SF-36 (PF, SF) | SF-36 (PF): MT 34.1 to 56.1, PE 37.3 to 48.8;  SF-36 (SF): MT 35.4 to 51.7, PE 35.4 to 49.0 | Both MT and PE significantly reduced phantom limb pain and improved quality of life and psychological status, with MT showing significantly greater efficacy (p < 0.05). |

TK: Thoracic kyphosis; PNS: Peripheral nerve stimulation; PGIC: Patient Global Impression of Change; MT: Mirror Therapy; PE: Phantom Exercise; SF: Social functioning

Supplementary Table 2. Pain & Functional Assessments and Outcomes (Continued)

| Author | Pain type | Pain  Assessments | Pain Outcomes | Functional Assessments | Functional Outcomes | Study Summary |
| --- | --- | --- | --- | --- | --- | --- |
| Leijendekkers et al., 2019 | RLP, LBP | Self-reported; NRS (Scale: 0–10) | Back pain: 0.2 ± 0.3 at 6 months and 12 months; Stump pain: 3.2 ± 2.8 at 6 months, 2.1 ± 2.7 at 12 months | TUG, 6MWT, Medicare Functional Classification (K0–K4), SIGAM-WAP grade, walking distance (self-reported), mobility aid use | Hip abductor strength increased by 0.16 ± 0.03 (23%); Prosthesis wearing time median increased from 81 to 100; TUG improved by −1.9 ± 0.7 seconds (17%); 6MWT increased by 25 ± 12 meters (8%); Daily walking distance increased from 400 to 1900 meters; Q-TFA Global Score increased by 25 ± 4 (54%) | No significant change in back pain intensity (p = 0.437); Functional improvements occurred despite persistent stump pain; Back pain frequency decreased in non-wheelchair users but increased in wheelchair users |
| Spahn et al., 2019 | RLP | NRS (Scale: 0–10) | Pre-amputation pain: 4 ± 2; Post-amputation pain: 1 ± 1 | FSST; 3D gait analysis: velocity, cadence, stride length, step width; self-reported walking endurance | FSST: 12 to 5s; Velocity: 108 to 142cm/s; Stride length: 129 to 154cm; Cadence: 101 to 111steps/min; Step width: 16 to 12cm; Walking tolerance: 1 mile to 7miles | Pain reduction was accompanied by significant improvements in walking tolerance (p = 0.003) and gait parameters such as velocity (p < 0.001), stride length (p < 0.001), cadence (p = 0.008), and step width (p = 0.004). |
| Geertzen et al., 2020 | PLP, RLP | NRS (Scale: 0–10); TAPES-R | RLP (n=23): little 13%, annoying 22%, alarming 35%, terrible 26%, unbearable 4%. | Self-reported mobility improvements; prosthesis use (hours/day); K-levels (converted from TAPES-R), WHOQOL-BREF | 77% reported important improvement in mobility; 73% fitted with a prosthesis; 40% used prosthesis ≥8 hours/day; WHOQOL-BREF physical domain: 12.7 ± 3.4 (lower than Dutch norm 15.2 ± 2.6) | Worst pain last week decreased significantly (p < 0.001). No significant changes in WHOQOL-BREF scores or psychological distress (p > 0.05). Most reported improved mobility with pain reduction. |

FSST: Four Square Step Test; TAPES-R: Trinity Amputation and Prosthesis Experience Scales – Revised; WHOQOL-BREF: World Health Organization Quality of Life-BREF

Supplementary Table 2. Pain & Functional Assessments and Outcomes (Continued)

| Author | Pain type | Pain  Assessments | Pain Outcomes | Functional Assessments | Functional Outcomes | Study Summary |
| --- | --- | --- | --- | --- | --- | --- |
| Mahon et al., 2020 | LBP | Self-reported; VAS (Scale: 0–100) | BK group: LBP increased at 6 and 12 months (50%); VAS while walking highest at 4 months (58%). AK group: LBP peaked at 4 months (33%) and dropped to 25% at 12 months; | Motion capture for 3D trunk and pelvis kinematics; Trunk and pelvis ROM; CRP analysis; walking at self-selected and controlled velocities | Trunk and pelvis ROM decreased over the first year, with smaller pelvis ROM reduction in BK than AK. Trunk-pelvis coordination (CRP) increased over time. BK showed lower sagittal ROM and higher frontal coordination than AK. | Significant time-group interactions for sagittal CRP (p=0.005), transverse CRP (p=0.009), and trunk ROM (p=0.013). LBP trends aligned with trunk-pelvis coordination changes; BK showed increasing LBP with rising CRP, AK showed decreasing LBP despite rising CRP. |
| Pleus et al., 2020 | PLP | VAS (Scale: 0–10); Qualitative description (prickling, painful, etc.) | PLP was very low at baseline (mean VAS ~0.3–0.4). Electrical stimulation did not significantly alter pain levels (~0.4 ± 1.3 ON vs 0.3 ± 0.9 OFF). | Gait parameters including step width (cm), stance and swing phase percentages, and self-selected walking speed (m/s) | Step width: 18.4 ± 2.7 cm (ON) vs 18.7 ± 2.5 cm (OFF); Stance phase: 60.2% ± 3.6% (ON) vs 59.9% ± 3.5% (OFF); Swing phase: 39.8% ± 3.6% (ON) vs 40.1% ± 3.5% (OFF); Walking speed: 1.1 ± 0.2 m/s (ON and OFF) | No significant changes in gait parameters or phantom pain with acute electrical stimulation, suggesting safety but no immediate functional or pain benefit (p-values > 0.05 except stance and swing phases). |
| Damiani et al., 2021 | PLP, RLP | NRS (Scale: 0–10) | Pain reported by 67.9% of subjects. Mean NRS = 6.78 ± 2.13. Phantom limb pain (40.5%), stump pain (14.6%), both (44.9%). | WHS for community ambulation | 27.2% unable to walk in the community (WHS 1–3). 79.8% regularly use prosthesis. 49.7% worked before amputation; 25.5% currently working | Pain intensity showed no significant correlation with community ambulation recovery (r = –0.120, p = 0.086; regression p = 0.338) Positive predictors of community ambulation: Prosthesis use (p < 0.001); Current working status (p = 0.009); Time since last amputation surgery (p = 0.014) |

WHS: Walking Handicap Scale

Supplementary Table 2. Pain & Functional Assessments and Outcomes (Continued)

| Author | Pain type | Pain  Assessments | Pain Outcomes | Functional Assessments | Functional Outcomes | Study Summary |
| --- | --- | --- | --- | --- | --- | --- |
| Fernandes et al., 2021 | RLP, LBP, ILP | NRS (Scale: 0–10); Pain drawing (42 body regions) | Worst pain: Patients 5 (5), Controls 0 (2); Mean pain: Patients 3 (3), Controls 0 (1); Current pain: Patients 1 (3), Controls 0 (1); Painful regions: Patients 2.5 (4), Controls 0 (3) | 6MWT, 30-Second CST, TESS; also ROM and isometric muscle strength using a dynamometer | Patients vs. Controls: 6MWT 499 vs. 607 m; CST 12 vs. 18 reps; TESS 78 vs. 100; Hip flexion ROM 113° vs. 130°; Knee flexion ROM 113° vs. 146°; Knee extension strength 0.9 vs. 2.1 Nm/kg; Hip abduction strength 1.1 vs. 1.9 Nm/kg | Higher pain and more painful regions correlated with lower physical function (Rho ≈ -0.4 to -0.54, p ≤ 0.03). Muscle strength positively correlated with physical function (Rho = 0.40–0.51, p < 0.05). Both pain and strength were linked to quality of life (p < 0.05). |
| Linden and Kiekens, 2021 | PLP, RLP | NRS (Scale: 0–10); Survey based on GQPLA and WHOQOL-BREF | 69% reported pain reduction after amputation; 13 patients experienced RLP (5 mild, 4 moderate, 4 severe); 14 patients had PLP (4 mild, 6 moderate, 4 severe); | Walking distance (<100m to >5km); prosthesis use hours/day (0 to >8h/day); Patient-reported changes in daily living activities | 11 of 16 patients reported improved mobility post-amputation; Most patients used prostheses daily (8 >8 h/day, 4 4–8 h/day, 2 <4 h/day); Walking distances increased for many (e.g., more patients walking >500 m after amputation); | Most patients were satisfied with the amputation (81%), and 69% reported improved pain and mobility. PLP persisted in most but was usually mild. |
| Major Extremity Trauma Research Consortium (METRC), 2021 | n.a. | BPI – severity and interference scores | After limb salvage: 3.58 (severity) and 3.83 (interference) | SMFA | SMFA mobility score after limb salvage was 38.62, with an improvement of 6.99 following amputation (95% CI: 2.78–11.04). | All pain and function improvements are statistically significant (p < 0.05); SMFA mobility improvements with amputation are both statistically and clinically meaningful |

CST: Chair-Stand Test; SMFA: Short Musculoskeletal Function Assessment

Supplementary Table 2. Pain & Functional Assessments and Outcomes (Continued)

| Author | Pain type | Pain  Assessments | Pain Outcomes | Functional Assessments | Functional Outcomes | Study Summary |
| --- | --- | --- | --- | --- | --- | --- |
| Srinivasan et al., 2021 | RLP, PLP | PROMIS Pain Interference 6a Survey;  NRS (Scale: 0–10); BPI | PROMIS Pain Interference: Reduced from 59.2 (pre-op) to 51.3 (post-op); BPI Pain Severity: Reduced from 5.6 to 1.5; BPI Pain Interference: Reduced from 6.8 to 2.1; NRS (PLP): Reduced by ~70% (from ~6 to ~2) | EMG for motor control; phantom limb ROM; positional discrimination task; ultrasound for muscle dynamics; self-report on prosthesis use and mobility | ROM percepts were greater in the AMI group postoperatively. Greater muscle fascicle strains were observed in the AMI group. AMI subjects demonstrated more distinct and precise muscle activation levels with less variance during motor tasks. | AMI subjects reported significantly less pain (p < 0.005) and greater phantom sensations (p < 0.05), which correlated with improved neuromuscular control (p < 0.05) and proprioceptive feedback. |
| Trouillez et al., 2021 | Neuropathic pain | VAS (Scale: 0–10);  DN4 | AK group VAS 2.7 ± 2.2, Arthrodesis group VAS 3.1 ± 3.3; AK group DN4 1.5 ± 2.1, Arthrodesis group 2.6 ± 2.9 | PPMS (Scale: 0–9); SF-36 Health Survey (physical function domain) | AK group PPMS: 5.2 ± 1.7, Arthrodesis group PPMS: 4.6 ± 1.4; AK group SF-36: 30.9 ± 15.6, Arthrodesis group SF-36: 26.9 ± 17.0 | VAS, DN4, PPMS, SF-36 scores all significantly better in AK group than arthrodesis (p < 0.001) |
| Younesian et al., 2021 | n.a. | VAS (Scale: 0–10) | VAS before 6MWT: 0.55 ± 1.57; after 6MWT: 2.95 ± 3.10 | IMUs for cadence, speed, and stance ratio; Polar HR monitor; total 6MWT distance also recorded | Distance walked: 322 ± 128 m; HR rose from 62% ± 11% to 70% ± 12% HRmax; Cadence dropped from ~100 to ~96–99 steps/min; Speed declined from 1.06 to ~0.99 m/s; Stance ratio increased slightly from 62% to 64% of gait cycle | Post-test pain increase linked to reduced cadence and speed due to fatigue. HR rise shows increased effort despite worsening gait. Changes in HR, cadence, speed, and stance ratio were statistically significant (p < 0.05 to < 0.001). |

PROMIS: Patient-Reported Outcomes Measurement Information System; AMI: Agonist–Antagonist Myoneural Interface; IMU: Inertial Measurement Units; HR: Heart Rate

Supplementary Table 2. Pain & Functional Assessments and Outcomes (Continued)

| Author | Pain type | Pain  Assessments | Pain Outcomes | Functional Assessments | Functional Outcomes | Study Summary |
| --- | --- | --- | --- | --- | --- | --- |
| Zaheer et al., 2021 | PLP | VAS (Scale: 0–10) | Experimental group VAS at 4 weeks: 2.25 ± 0.62; Control group VAS at 4 weeks: 3.58 ± 1.24 | AMP | Experimental group AMP at 4 weeks: 25.92 ± 4.12; Control group AMP at 4 weeks: 24.33 ± 4.61 | Phantom exercises added to mirror therapy and routine physiotherapy significantly improved pain (p = 0.003) and bodily pain-related quality of life (p = 0.012) but did not significantly improve mobility (p = 0.385). |
| Atar et al., 2022 | RLP, LBP, ILP | VAS (Scale: 0-10) | NAA vs. AHA: RLP 2.6 ± 2.8 vs. 4.0 ± 3.5, ILP 1.4 ± 2.1 vs. 2.0 ± 2.2, LBP 1.8 ± 2.5 vs. 2.5 ± 2.7 | 6MWT, Houghton Score, SF-36 (QoL), Likert scale for terrain navigation difficulty | 6MWT distance (m): NAA 557.5 ± 67.7, AHA 540.4 ± 62.3; SF-36 Physical Functioning: NAA 68.5 ± 28.0, AHA 68.8 ± 26.4; Difficulty descending ramps (Likert scale): Higher difficulty in NAA group | No significant pain or functional differences between NAA and AHA groups (p > 0.05). In AHA group, BMI correlated with low back pain (r=0.45, p=0.04), stump length correlated with residual and intact limb pain (r=0.50 and 0.55, p<0.05). In NAA group, socket comfort correlated with foot satisfaction (r=0.56, p=0.009). |
| Deldar et al., 2022 | n.a. | NRS (Scale: 0-10), PROMIS Pain Interference and Pain Intensity | Best pain (24h): 1.7 ± 2.1; Worst pain (24h): 2.8 ± 2.5; PROMIS Pain Interference: 52.5 ± 8.4; PROMIS Pain Intensity: 50.4 ± 10.7 | LEFS | LEFS score: 47.1 ± 14.7 (TMA success: 49.1 ± 14.0, Higher-level amputation: 39.1 ± 15.7) Maximal function (%): 58.9 ± 18.4 | Higher-level amputation patients reported significantly higher current pain (p = 0.016) and tended to have lower functional scores (p = 0.068). |

PROMIS: Patient-Reported Outcomes Measurement Information System; AMI: Agonist–Antagonist Myoneural Interface; IMU: Inertial Measurement Units; HR: Heart Rate; NAA: Non-articulating ankle; AHA: Articulating hydraulic ankle, LEFS: Lower Extremity Functional Scale

Supplementary Table 2. Pain & Functional Assessments and Outcomes (Continued)

| Author | Pain type | Pain  Assessments | Pain Outcomes | Functional Assessments | Functional Outcomes | Study Summary |
| --- | --- | --- | --- | --- | --- | --- |
| Donati et al., 2022 | PLP, RLP | PEQ (pain) | 67% had PLP, 62.3% PLS, and 52.2% RLP. Mean pain scores: PLS = 4.1, PLP = 3.5, RLP = 3.4 (scale 1–7). | Houghton Scale (Scale: 0-12; prosthesis use: duration, context, aid use, confidence in different terrains) | BK had the highest prosthesis use (mean Houghton = 9.2), followed by AK (8.2), and hemipelvectomy (4.0). Confidence on uneven terrain declined with age | While lower levels of amputation and younger age predict higher prosthetic use, chronic pain syndromes (PLP, PLS, RLP) were not significantly associated with decreased prosthesis use in this sample. |
| Ernstsson et al., 2022 | PLP, RLP | EQ-5D-3L and EQ-5D-5L (pain/discomfort dimension); Self-report | 67% had PLP, 50% had RLP; 70% (EQ-5D-3L) and 75% (EQ-5D-5L) reported problems in pain/discomfort dimension | LCI-5; Prosthetic Use Score; EQ-5D dimensions: mobility, self-care, usual activities | LCI-5 median: 28 (IQR 12–43); Prosthetic use median: 32 (IQR 6–51); EQ-5D mobility problems: 78% (3L), 75% (5L); Usual activities problems: 61% (3L), 74% (5L) | EQ-5D-5L correlated stronger than 3L (p < 0.01). LCI-5 moderately to strongly correlated with EQ-5D domains (p < 0.01). PLP and RLP moderately correlated with EQ-5D pain (p < 0.01). Higher-level amputations had lower EQ-5D-5L scores (p = 0.046). |
| Kannenberg et al., 2022 | RLP, LBP, ILP | NRS; PROMIS Pain Interference Short Form (6a) | PwrAF users reported lower sound knee pain (median 1) than passive foot users (median 1.5); in moderate/severe cases, PwrAF further reduced sound (3 vs. 5.5) and amputated knee pain (3 vs. 6). | PLUS-M | PwrAF users had higher mobility scores (54.9 ± 6.0) than passive foot users (50.3 ± 7.8). Among those with moderate/severe knee pain on passive feet, the difference was larger (52.8 ± 3.9 vs 40.8 ± 4.6). | Reduced sound and amputated knee pain with powered foot correlated with higher mobility scores (p < 0.05). No significant difference in pain interference was observed (p > 0.05). |

PEQ: Prosthetic Evaluation Questionnaire; PwrAF: Powered ankle-foot; PLUS-M: Prosthetic Limb Users’ Survey of Mobility; SF: Symmetry Function

Supplementary Table 2. Pain & Functional Assessments and Outcomes (Continued)

| Author | Pain type | Pain  Assessments | Pain Outcomes | Functional Assessments | Functional Outcomes | Study Summary |
| --- | --- | --- | --- | --- | --- | --- |
| Lu et al., 2022 | PLP, RLP | NRS (Scale: 0-10); PROMIS Pain Intensity, Interference | NI vs. Control: Lower worst PLP (3.5 vs. 4.9) and RLP (2.6 vs. 4.4); reduced PROMIS pain intensity and interference scores for both PLP and RLP. | Neuro-QoL Lower Extremity Function (Mobility) Short Form | NI group: 45.4 ± 3.6, Control group: 41.9 ± 5.2 | NI group showed significant improvements in RLP severity (p = 0.035), overall pain intensity (PLP p = 0.018, RLP p = 0.014), pain interference for PLP (p = 0.015), pain behaviour for RLP (p = 0.025), and functional outcomes (p = 0.032), while no significant difference was found in worst PLP (p = 0.298). |
| Kar and Kutlu, 2023 | PLP, RLP | SF-36 pain subscale | SF-36 pain score: 48.13 ± 28.33 | SF-36 PF; Qualitative questions on mobility and prosthesis use; Amputee Body Image Scale(ABIS) | SF-36 physical function score: 30.11 ± 20.00, social function: 36.66 ± 27.45, physical roles: 18.33 ± 30.03;  ABIS: 60.1 ± 19.62 | Poorer body image was significantly associated with lower quality of life and function, showing negative correlations with physical function (r = -0.59, p < 0.001), social function (r = -0.55, p < 0.001), and pain (r = -0.43, p = 0.01). |
| Seth et al., 2023 | RLP, LBP, ILP | NRS (Scale: 0–10 scale) | Median average pain intensity AK 0.75 (IQR 0–1.4), BK 0 (IQR 0–1.8); pain in 1+ regions: AK 57.7%, BK 49.1% | PLUS-M, Activities-Specific Balance Confidence (ABC), Functional Reach Test (FRT), mFSST | ABC median AK 53, BK 57; PLUS-M T-score AK 55.8, BK 59.6; FRT prosthetic side AK 28.2 cm, BK 31.0 cm | Pain in all three regions increased recurrent fall odds 6.5× (p = 0.045); each 1-point rise in ABC and PLUS-M scores reduced odds by 7.3% (p = 0.017) and 9.4% (p = 0.020), respectively |
| Wijekoon et al., 2023 | PLP, RLP, LBP | Self-reported | PLP prevalence: 77.6%, Back pain prevalence: 69.4%, Knee pain prevalence: 20% | 2MWT, TUG, K-level classification | 2MWT distance: 113.6 ± 14.8 m (veterans) vs. 150.8 ± 11.9 m (controls); TUG time: 10.6 ± 1.8 s (veterans) vs. 7.2 ± 0.9 s (controls); Functional level: 71.8% veterans classified as K3 | Veterans with LLA had higher physical comorbidities (p < 0.001–0.021), lower functional mobility, and greater fall risk than controls (p < 0.001). Functional differences between BK and AK amputees were significant only for TUG (p < 0.001). |

Supplementary Table 2. Pain & Functional Assessments and Outcomes (Continued)

| Author | Pain type | Pain  Assessments | Pain Outcomes | Functional Assessments | Functional Outcomes | Study Summary |
| --- | --- | --- | --- | --- | --- | --- |
| Berger et al., 2024 | RLP | Self-reported | RLP limited physiotherapy completion in 24% of patients on postoperative day 1 | Basic Amputee Mobility Score | Limitations were mainly due to fatigue (44%) and fear of mobilization (33%). Physiotherapy completion was 63%, limited by fatigue and RLP. | RLP (p = 0.02, 0.03), fatigue (p = 0.002, 0.005), and fear of mobilization (p = 0.004, 0.008) significantly limited early mobility and physiotherapy completion, highlighting key targets for acute rehabilitation. |
| Butowicz et al., 2024 | LBP | VAS (Scale: 0-10) | LBP group VAS: 2±1, No-pain group VAS: 0±1. | Kinematics and kinetics, EMG-informed musculoskeletal modeling (OpenSim), lumbar spinal loads (L5-S1) | LBP group’s ipsilateral shear forces increased more with speed (~1.33 m/s) than no-LBP (~1.35 m/s), who had larger contralateral shear forces; EMG showed higher external oblique and psoas activation in no-LBP. | Pain × speed affected shear forces (ipsilateral p=0.023; contralateral p=0.047, 0.008). No-pain group showed higher external oblique (p=0.039) and prosthetic psoas activation (p≤0.025), with pain × speed interaction in internal oblique (p=0.003). |
| Çalışkan et al., 2024 | LBP | VAS (Scale: 0-10) | LBP rose after 6MWT but fell below baseline 30 minutes post-application, with greater reduction in the KT group (KT: 1.47 ± 0.91 to 0.87 ± 0.74; Sham: 1.57 ± 0.85 to 1.36 ± 0.63). | 6MWT, standing balance using a force platform (MatScan): AP and ML sway with eyes open/closed | KT group walked farther (494 ± 66 m) than control (403 ± 63 m). AP sway rose post-6MWT in both groups but normalized after 30 minutes only with KT. Eyes-closed AP and ML sway decreased only in KT group after 30 minutes. | Pain reduction 30 minutes post-KT was greater in the experimental group (p = 0.016). AP sway improved eyes open (p = 0.010) and decreased eyes closed (p = 0.032), with ML sway also reduced eyes closed (p = 0.010). |

KT: Kinesiology taping; AP: Anterior-posterior; ML: Mediolateral

Supplementary Table 2. Pain & Functional Assessments and Outcomes (Continued)

| Author | Pain type | Pain  Assessments | Pain Outcomes | Functional Assessments | Functional Outcomes | Study Summary |
| --- | --- | --- | --- | --- | --- | --- |
| Gaffney et al., 2024 | LBP | Modified-ODI | mODI: 32.4 ± 19.9 to 15.4 ± 18.2 | Whole-body motion capture during overground walking; lumbopelvic ROM; CRP coordination analysis. | Sagittal plane pelvis and trunk ROM decreased post-implantation. In-phase sagittal lumbopelvic coordination increased during amputated limb stance, while anti-phase transverse coordination increased during intact limb loading response. | Reduced LBP disability (mODI p = 0.013) coincided with decreased sagittal trunk and pelvis ROM (p ≤ 0.001) and improved lumbopelvic coordination, shown by CRP changes in sagittal and transverse planes (p < 0.001 and p = 0.029), indicating less compensatory movement. |
| Hotta et al., 2024 | n.a. | BPI – Severity and Interference subscales | Amputee group BPI: 1.35 to 0.77 | FIM, baropodometry (plantar pressure metrics); ROM and strength testing (manual dynamometry) | FIM: 122.53 to 122.33, Plantar pressure lateral velocity 0.27 to 0.01 | Amputees showed significant pain interference reduction (p < 0.05), however, FIM scores remained stable (p > 0.05), suggesting pain improvement didn’t immediately enhance functional independence. |
| Pousett et al., 2024 | n.a. | NRS (Scale: 0-10) | Pain scores decreased from 2.7 ± 1.9 at initial rehab baseline to 1.0 ± 1.0 at discharge, rose to 3.7 ± 3.2 at replacement socket evaluation, then dropped to 1.4 ± 1.7 at definitive fitting. | 2MWT – walking distance (metres), PLUS-M | 2MWT distance increased from 68.4 ± 32.4 m at baseline to 107.4 ± 32.3 m at discharge, then to ~130 m during socket replacement. PLUS-M scores rose from 38.9 ± 19.4 to 53.4 ± 8.4 at discharge, remaining stable thereafter. | Pain significantly decreased during rehab and replacement (p < 0.05). Functional mobility (2MWT, PLUS-M) improved significantly during rehab (p < 0.001) with smaller changes in replacement. No measure exceeded the Minimal Detectable Change, showing individual variability. |

Supplementary Table 2. Pain & Functional Assessments and Outcomes (Continued)

| Author | Pain type | Pain  Assessments | Pain Outcomes | Functional Assessments | Functional Outcomes | Study Summary |
| --- | --- | --- | --- | --- | --- | --- |
| Schnetz et al., 2024 | Neuropathic pain | NRS (Scale: 0–10), WOMAC pain score | NRS pain: 2.70 to 1.91; WOMAC pain score: 5.26 | WOMAC activity score, SF-12, EQ-5D-5 Level questionnaire, AAS; PPMS | WOMAC activity 43.21, function 18.13, SF-12 physical health 29.06. Only 45.7% fitted with prosthesis; 50% walked with it, 8.3% unaided. After rehab, 19.6% walked >500 m. | AK group showed limited function and mobility compared to arthrodesis: WOMAC activity (p = 0.017), WOMAC function (p = 0.019), SF-12 physical health (p = 0.016), walking ability (p = 0.001), and walking distance over 500 m (p = 0.002). |
| Demofonti et al., 2025 | Neuropathic pain | NRS (Scale: 0-10), NPSI | NRS: BK 6→2, AK 0→0;  NPSI: BK 6→0, AK 0→0 | Motion capture system (Spatiotemporal, Symmetry index), instrumented insole (vGRF) | BK: peak vGRF from 96% → 143% BW, Step length symmetry (11% → 5%). AK: vGRF from 66% → 72% BW. Stance phase symmetry −4% → −2%. | BK showed reduced pain with improved gait (prosthetic loading, p<0.006; step length symmetry, p=0.001), while AK showed functional gains (prosthetic loading, p<0.006; stance symmetry, p<0.006) despite no pain change. |
| Fournier-Farley et al., 2025 | Neuropathic pain | NRS (Scale: 0-10) | NRS < 4 group : median score of 0,  NRS ≥ 4 group : median score of 7. | Prosthesis use, K-level, PDI, LCI-5 | Prosthesis use was high (9–12 h/day) across groups, walking aids were rarely used, median K-level was 2, and LCI-5 scores were similarly high (≈44–46) regardless of pain severity. | Prosthetic wear time ↓ with higher pain (p=0.033), Walker use ↔ lower pain (p=0.003), PDI ↑ with higher pain (p<0.0001), K-level (p=0.166), LCI-5 (p=0.151) |
| Parr et al., 2025 | LBP | NRS (Scale: 0-10) | LBP: 3.7 ±1.9 | IMU: STS time, acceleration magnitude, jerk variance (SI/AP/ML) | STS time: LBP 12.1 ± 3.8, no LBP 11.6 ± 2.9 s, The RMS of acceleration and variance of jerk were similar in the SI (p = 0.26) and AP (p = 0.56) | In the ML direction, variance of jerk for sit-to-stand (p =0.004) and stand-to-sit (p = 0.010), were greater in LBP group |

WOMAC: Western Ontario and McMaster Universities Osteoarthritis Index; AAS: Amputee Activity Score; NPSI: Neuropathic Pain Symptom Inventory; PDI: Pain Disability Index; STS: Sit to Stand; SI: Superior-Inferior; AP: Anterior-Posterior; ML: Medio-Lateral

Supplementary Table 2. Pain & Functional Assessments and Outcomes (Continued)

| Author | Pain type | Pain  Assessments | Pain Outcomes | Functional Assessments | Functional Outcomes | Study Summary |
| --- | --- | --- | --- | --- | --- | --- |
| Penasso et al., 2025 | PLP | VAS (Scale: 0-100) | Vibration group ΔVAS: 2.73 ± 12.36  Control group ΔVAS: -4.81 ± 16.02 | Gaitrite, TUG, 2MWT,10MWT, FSST, EQ-5D-3L, TAPES-R | Δ (mean ± SD), Vibration vs Control: gait speed -1.06 ± 14.73 vs 4.73 ± 11.69; AL stance time 0.32 ± 8.22 vs 1.97 ± 7.48; step length 0.24 ± 3.60 vs -0.86 ± 3.79; TUG 0.21 ± 1.05 vs 0.44 ± 0.84; FSST 0.34 ± 0.53 vs 0.07 ± 0.86; 2MWT 5.17 ± 13.95 vs 4.76 ± 19.61. | In the vibrotactile feedback trial, neither pain (VAS: ΔV = 2.73 ± 12.36; ΔC = −4.81 ± 16.02) nor functional outcomes (gait speed, stance time, step length, TUG, FSST, 2-min walk) showed significant group effects (all p > 0.05), indicating limited interplay between pain and function |
| Rierola-Fochs et al., 2025 | PLP | Short-Form MPQ (SF-MPQ) | PLP ↓ 5.85→3.00 (p = .005, r = 0.89), clinically meaningful (>2 cm). Control: no change | FIM, EQ-5D-5L for quality of life | No statistically significant changes in FIM scores (mean change ~1.2 pts). | Home-based Graded Motor Imagery showed meaningful reductions in PLP, highlighting the interplay between pain relief and functional improvement |
| Shaw et al., 2025 | PLP, RLP, LBP, ILP | NRS, PROMIS-GH. Q-TFA | (Baseline) NRS: BK 4.9 ±2.5, AK 3.1 ±2.6; PROMIS: AK ΔRLP = −21.2 ± 1.5, AK ΔLBP = −20.8 ± 1.9; BK ΔRLP = −21.8 ± 1.4 | Q-TFA (QoL related questions) | QoL gains: RLP (walking/standing) – AK: +21.3 ± 1.5, BK: +22.1 ± 1.5; AK – LBP: +21.1 ± 1.9, PLP: +21.0 ± 1.5, RLP (no prosthesis): +20.7 ± 1.7, ILP: +20.8 ± 1.6; BK – RLP (no prosthesis): +20.8 ± 1.3. | RLP during walking/standing improved in AK and BK with parallel QoL gains (p<0.001). Back pain improved only in AK (p=0.02). PLP, RLP without prosthesis, and contralateral pain showed no reduction (p≥0.05), though QoL gains persisted (p=0.003–0.04) |
