# Supplementary material for: Assessment of pain and functional outcomes after lower limb amputation: a scoping review
Source: BMJ Open. 2026 Mar 10;16(3):e110319. doi: 10.1136/bmjopen-2025-110319 (PMC12983873; doi:10.1136/bmjopen-2025-110319)
Supplement: Supplementary file 4 [file bmjopen-2025-110319.reviewer_comments.pdf]

PEER REVIEW HISTORY

BMJ Open publishes all reviews undertaken for accepted manuscripts. Reviewers are asked to complete a checklist review form and are provided with free text boxes to elaborate on their assessment. These free text comments are reproduced below.

ARTICLE DETAILS

Title (Provisional)

Assessment of Pain and Functional Outcomes After Lower Limb Amputation: A Scoping Review

Authors

Kim, Jin Min; Balthazaar, Shane JT; Alsayed, Khalid; Nightingale, Thomas; Falla, Deborah; Yeo, Sang-Hoon; Ding, Ziyun

---

VERSION 1 - REVIEW

**Reviewer**	**1**
**Name**	**Ramadanov, Nikolai**
**Affiliation**	**University Hospital Brandenburg/Havel Brandenburg Medical School**
**Date**	**22-Oct-2025**
**COI**	**None**

---

1. The manuscript provides a valuable synthesis of pain and function assessment after lower limb amputation (LLA). However, the stated novelty could be made clearer — previous narrative reviews have covered related ground. The authors should delineate more explicitly what distinguishes this review.

2. The rationale for including low back pain (LBP) alongside amputation-specific pain (PLP, RLP) needs stronger justification. LBP may represent a secondary musculoskeletal adaptation rather than a direct post-amputation pain type. Clarify the conceptual boundary to avoid conflating primary and compensatory pain mechanisms.

3. While the search is broad and databases are appropriate, the exact PubMed/Embase syntax is not presented in the main text. A concise example search line should be included in the Methods for transparency and reproducibility.

4. Although scoping reviews do not require formal risk-of-bias assessment, BMJ Open often expects at least a qualitative comment on methodological quality. A brief overview of the typical study quality would strengthen the interpretability of findings.

5. The Results section is rich but somewhat descriptive and repetitive. Consider condensing overlapping subsections and highlighting the main patterns in clearer summary tables or a conceptual map.

6. The manuscript repeatedly notes heterogeneity in assessment tools but does not attempt any descriptive quantification. Adding basic frequency statistics would substantiate these statements.

7. The link between pain type and specific functional impairment remains narrative. A simple cross-tabulation or matrix summarising the main reported relationships could improve clarity and scientific value.

8. The Discussion appropriately mentions ISB guidelines, but the recommendation remains general. The authors could be more specific - e.g., suggest adoption of unified marker sets, reporting conventions, or use of open datasets to improve reproducibility across biomechanics studies.

9. The text is well written but overly long in several theoretical sections. The discussion on mechanistic parallels with other diseases could be shortened or moved to Supplementary Material to maintain focus on LLA.

10. The conclusions are balanced but could be sharpened to include concrete research priorities - such as longitudinal monitoring using IMUs, multimodal pain - function protocols, or integrating objective biomechanical markers into rehabilitation trials.

**Reviewer**	**2**
**Name**	**Sirignano, Pasqualino**
**Affiliation**	**University of Rome La Sapienza**
**Date**	**10-Dec-2025**
**COI**	**None**

I have read with great attention and interest the paper entitled "Assessment of Pain and Functional Outcomes After Lower Limb Amputation: A Scoping Review".

The topic is relevant and the study addresses an important gap in the current literature. The methods are generally appropriate, and the structure is coherent. However, the manuscript suffers from a major problem of excessive length and would benefit from substantial condensation. Several sections—particularly the Introduction and Discussion—are unnecessarily expansive, repeating concepts already well established and diluting the central message of the study. Streamlining the background and focusing more strictly on the novel findings would considerably improve readability and impact.

In addition, the presentation of results could be made more concise. Some tables and figures appear redundant or present highly detailed information that is not essential for understanding the main conclusions. A more selective presentation would enhance clarity.

VERSION 1 - AUTHOR RESPONSE

Reviewer: 1
Dr. Nikolai Ramadanov, University Hospital Brandenburg/Havel Brandenburg Medical School, Brandenburg Medical School Theodor Fontane
Comments to the Author:
1. The manuscript provides a valuable synthesis of pain and function assessment after lower limb amputation (LLA). However, the stated novelty could be made clearer — previous narrative reviews have covered related ground. The authors should delineate more explicitly what distinguishes this review.
We thank the reviewer for this comment and agree that previous narrative and systematic reviews have addressed post-amputation pain and functional outcomes following lower limb amputation. To clarify the novelty of the present review, we have revised the Introduction to explicitly acknowledge these prior reviews and to delineate their scope and limitations.

Specifically, we now highlight that existing reviews across different pain phenotypes (phantom limb pain, residual limb pain, and secondary musculoskeletal pain) have acknowledged potential functional implications, but have largely discussed pain–function relationships descriptively rather than systematically synthesising evidence. We further clarify that functional outcomes have not been integrated across pain phenotypes or assessment approaches, and that this lack of integration is also evident in primary research.

These revisions more clearly distinguish the present scoping review by emphasising its focus on mapping pain–function relationships across pain phenotypes and identifying gaps that hinder integrated evaluation. By doing so, this review moves beyond descriptive summaries to provide a structured overview of pain–function relationships across pain phenotypes and functional domains.

(page 5, line6-21)

2. The rationale for including low back pain (LBP) alongside amputation-specific pain (PLP, RLP) needs stronger justification. LBP may represent a secondary musculoskeletal adaptation rather than a direct post-amputation pain type. Clarify the conceptual boundary to avoid conflating primary and compensatory pain mechanisms.

We agree that low back pain differs mechanistically from amputation-specific pain such as phantom limb pain and residual limb pain and primarily represents a secondary musculoskeletal condition arising from compensatory gait mechanics following lower limb amputation.

To address this, we have revised the Introduction to explicitly clarify this conceptual boundary. We now state that low back pain is included in the present review not as an amputation-specific pain, but due to its high prevalence following lower limb amputation and its established relevance to functional mobility and rehabilitation outcomes. This clarification avoids conflation of primary and compensatory pain mechanisms while maintaining a function-oriented framework.

(page 5, line2-5)

3. While the search is broad and databases are appropriate, the exact PubMed/Embase syntax is not presented in the main text. A concise example search line should be included in the Methods for transparency and reproducibility.

The detailed search strategy, including an example Medline search line, has now been provided as Supplementary Material, as referenced in the Methods section, to improve transparency and reproducibility. (Supplementary Material 1)

4. Although scoping reviews do not require formal risk-of-bias assessment, BMJ Open often expects at least a qualitative comment on methodological quality. A brief overview of the typical study quality would strengthen the interpretability of findings.

We thank the reviewer for this helpful suggestion. The Discussion has been revised to include a concise qualitative overview of the methodological characteristics of the included studies, highlighting common study designs, sample size characteristics, and key methodological limitations. (page 17, line16-22)

5. The Results section is rich but somewhat descriptive and repetitive. Consider condensing overlapping subsections and highlighting the main patterns in clearer summary tables or a conceptual map.
In response to this comment, we have revised the Results section to reduce repetition and highlight key patterns more clearly. Pain assessment methods and functional outcome measures were systematically classified, and their co-occurrence across studies is now synthesised using a heatmap that provides a concise visual summary. Based on this synthesis, the description of pain type–specific functional outcomes was streamlined to emphasise the main reported associations while avoiding redundant detail. (page 12- 16, Figure file)

6. The manuscript repeatedly notes heterogeneity in assessment tools but does not attempt any descriptive quantification. Adding basic frequency statistics would substantiate these statements.
We have clarified the use of descriptive frequency statistics in the Results to quantify heterogeneity in pain types, pain assessment tools, and functional outcome measures. Counts and frequencies are now explicitly reported in the text and figures to substantiate statements regarding methodological heterogeneity.

7. The link between pain type and specific functional impairment remains narrative. A simple cross-tabulation or matrix summarising the main reported relationships could improve clarity and scientific value.
In response to this comment, we have revised the Results by introducing a matrix-style summary figure (Figure 4) that cross-tabulates post-amputation pain phenotypes with key functional impairment domains. This visual synthesis summarises the main reported pain–function relationships across studies and facilitates comparison across pain types. (Figure 4)

8. The Discussion appropriately mentions ISB guidelines, but the recommendation remains general. The authors could be more specific - e.g., suggest adoption of unified marker sets, reporting conventions, or use of open datasets to improve reproducibility across biomechanics studies.
In response to this comment, the Discussion has been revised to provide more specific

methodological recommendations, including adoption of unified marker sets, clear reporting of coordinate system definitions and joint rotation sequences, and transparent documentation of data processing procedures in line with ISB guidance. The value of shared or openly available datasets for improving reproducibility is also highlighted. (page 20, line17-page 21,line10)

9. The text is well written but overly long in several theoretical sections. The discussion on mechanistic parallels with other diseases could be shortened or moved to Supplementary Material to maintain focus on LLA.
In response, the Discussion has been condensed and refocused to maintain a clear emphasis on LLA. The section discussing mechanistic parallels with other clinical populations has been streamlined to retain only core pain–function concepts directly relevant to interpreting the LLA literature, while avoiding extended disease-specific elaboration. Redundant examples have been removed, and the revised discussion now serves to contextualise the identified research gap in LLA rather than to provide a broad theoretical overview.

10. The conclusions are balanced but could be sharpened to include concrete research priorities - such as longitudinal monitoring using IMUs, multimodal pain - function protocols, or integrating objective biomechanical markers into rehabilitation trials.
The Conclusions have been revised to explicitly include concrete research priorities, including longitudinal mobility monitoring using IMUs, multimodal pain–function assessment protocols, and the integration of objective biomechanical markers into rehabilitation trials.(page23)

Reviewer: 2
Dr. Pasqualino Sirignano, University of Rome La Sapienza
Comments to the Author:
I have read with great attention and interest the paper entitled "Assessment of Pain and Functional Outcomes After Lower Limb Amputation: A Scoping Review".
The topic is relevant and the study addresses an important gap in the current literature. The methods are generally appropriate, and the structure is coherent.

However, the manuscript suffers from a major problem of excessive length and would benefit from substantial condensation. Several sections—particularly the Introduction and Discussion—are unnecessarily expansive, repeating concepts already well established and diluting the central message of the study. Streamlining the background and focusing more strictly on the novel findings would considerably improve readability and impact.

We agree that the manuscript benefited from greater conciseness and focus. Accordingly, we have substantially condensed both the Introduction and Discussion sections by removing repetitive background material and narrowing the theoretical framing to elements directly relevant to the aims of this scoping review. The revised text places greater emphasis on the novel synthesis of pain–function assessment patterns and the gaps identified across the literature.

In addition, the presentation of results could be made more concise. Some tables and figures appear redundant or present highly detailed information that is not essential for understanding the main conclusions. A more selective presentation would enhance clarity.
In response to this comment, detailed table previously included in the main text were removed and replaced with a heatmap to provide a more concise visual summary of the results. The heatmap also reports the frequency with which pain and functional assessments were used together across studies, thereby preserving the descriptive intent of the scoping review. In addition, pain type–

specific functional changes were summarised using a matrix-style figure to present key patterns more selectively and clearly. (Figure 3,4)

VERSION 2 - REVIEW

**Reviewer**	**1**
**Name**	**Ramadanov, Nikolai**
**Affiliation**	**University Hospital Brandenburg/Havel Brandenburg Medical School**
**Date**	**25-Jan-2026**
**COI**	

---

1. The primary objective of the study is generally clear; however, the clinical question would benefit from a more explicit framing, particularly with respect to whether the focus is superiority, non-inferiority, or equivalence between the compared interventions. Clearer alignment between the stated aim and the presented analyses would strengthen interpretability.

2. The included studies demonstrate variability in patient characteristics, surgical techniques, and follow-up duration. While this reflects real-world practice, the manuscript would benefit from a more explicit discussion of how this heterogeneity may influence the robustness and generalizability of the findings.

3. Multiple outcome measures are reported; however, their relative clinical importance is not always clear. A clearer distinction between primary and secondary outcomes, and between clinician-reported and patient-reported outcomes, would improve the clinical relevance of the results.

4. Several statistically significant differences are reported, but their practical implications are not consistently discussed. Greater emphasis on the magnitude and direction of effects, rather than statistical significance alone, would improve the clinical interpretation of the findings.

5. Although some limitations are acknowledged, the discussion would benefit from a more comprehensive consideration of potential biases inherent to the included study designs, including selection bias, performance bias, and outcome reporting bias, and how these may affect the conclusions.

6. The manuscript reports changes in patient-reported outcome measures but does not consistently contextualize these changes against established minimal clinically important differences (MCIDs). A dedicated discussion of whether observed improvements meet or exceed MCID thresholds is necessary to distinguish statistical from clinical relevance. The

authors should cite and incorporate available MCID methodology and interpretation frameworks (DOI: 10.1002/jeo2.70568).

VERSION 2 - AUTHOR RESPONSE

Reviewer: 1
Dr. Nikolai Ramadanov, University Hospital Brandenburg/Havel Brandenburg Medical School, Brandenburg Medical School Theodor Fontane
Comments to the Author:
1. The primary objective of the study is generally clear; however, the clinical question would benefit from a more explicit framing, particularly with respect to whether the focus is superiority, non-inferiority, or equivalence between the compared interventions. Clearer alignment between the stated aim and the presented analyses would strengthen interpretability.

Thank you for this comment. We have revised the manuscript to ensure clearer alignment between the stated aim and the analyses presented. Specifically, we have clarified in the Introduction that this study is a **scoping review**, designed to systematically map how pain and functional outcomes have been assessed and reported following lower limb amputation, rather than to evaluate comparative effectiveness. Consistent with this aim, the analyses are presented descriptively and synthesised narratively, without formal testing of superiority, non-inferiority, or equivalence. This alignment is further reinforced in the Discussion, where we explicitly state that the review is not intended to draw comparative conclusions between interventions. Together, these revisions strengthen interpretability and reduce the potential for misinterpretation.

In the discussion session, we added “In line with the objectives of a scoping review, this study was not designed to formally test superiority between specific assessments or interventions. Rather, our aim was to map how post-amputation pain and functional outcomes have been assessed and reported, and how the relationship between these domains has been described across the literature to date. Accordingly, the findings should be interpreted as an evidence map of current assessment approaches and reporting practices, rather than as comparative effectiveness conclusions.”

2. The included studies demonstrate variability in patient characteristics, surgical techniques, and follow-up duration. While this reflects real-world practice, the manuscript would benefit from a more explicit discussion of how this heterogeneity may influence the robustness and generalizability of the findings.

Thank you for this comment. We agree that heterogeneity in patient characteristics, surgical techniques, and follow-up duration is a key feature of the included literature and has important implications for both robustness and generalizability. To address this explicitly, we have expanded the Discussion to clarify how heterogeneity in pain types, outcome selection, and study design constrains interpretation and limits causal inference. We also emphasise that the findings should be interpreted as indicative patterns observed across heterogeneous clinical contexts, rather than as generalisable estimates applicable to specific patient subgroups or intervention pathways.

Specifically, in the revised manuscript, we added: “Heterogeneity in pain types, patient characteristics, outcome selection, and study design, together with limited longitudinal and mechanistic investigation, constrains interpretation and precludes firm causal inference. Consequently, the findings should be interpreted as indicative patterns observed across heterogeneous clinical contexts, rather than as generalisable estimates applicable to specific patient subgroups or intervention pathways.”

3. Multiple outcome measures are reported; however, their relative clinical importance is not always clear. A clearer distinction between primary and secondary outcomes, and between clinician-reported and patient-reported outcomes, would improve the clinical relevance of the results.

Thank you for this comment. We agree that distinguishing the clinical importance of different outcomes can be valuable. However, as this study was designed as a scoping review, our primary aim was not to define or rank outcomes according to their relative clinical importance, but **to examine how pain and functional outcomes have been linked and discussed across the existing literature in individuals with lower limb amputation, despite limited consistency across studies**. Accordingly, the Discussion focuses on describing patterns in outcome use and reporting, rather than assigning primary or secondary outcome status, to reflect prevailing assessment practices and improve interpretive transparency.

We have revised the manuscript by adding a dedicated **“Clinical Implications and Future Directions”** section. Specifically, we added “Across pain phenotypes, the available evidence suggests that post-amputation pain is associated with functional limitations across multiple domains. These associations are most commonly derived from outcomes that are treated as primary endpoints in the included studies, including patient-reported measures of pain severity, prosthetic use, and participation, alongside clinician-reported or instrument-based assessments of mobility and gait performance. Using these outcome categories, studies of PLP and RLP—despite receiving less detailed biomechanical investigation—consistently reported reduced prosthetic use, slower walking speed, impaired balance, and restrictions in daily activity and participation, largely based on patient-reported outcomes and clinical walking tests. In contrast, LBP has been examined more extensively using clinician-reported and instrumented biomechanical outcomes, demonstrating consistent associations with altered gait patterns, trunk and pelvic motion abnormalities, balance impairments, and reduced movement efficiency. Collectively, these patterns indicate that pain is not merely a coexisting symptom but appears to exert a measurable influence on functional performance after lower limb amputation, as reflected across both patient-reported and clinician-derived outcome domains.”

4. Several statistically significant differences are reported, but their practical implications are not consistently discussed. Greater emphasis on the magnitude and direction of effects, rather than statistical significance alone, would improve the clinical interpretation of the findings.

Thank you for this comment. We have revised the Discussion to place greater emphasis on the **direction and consistency of reported pain–function associations across studies**, rather than on statistical significance alone.

As this review was conducted as a scoping review, and given substantial heterogeneity in study design, outcome measures, statistical approaches, and reporting of effect sizes, it was not possible to consistently evaluate or compare the magnitude or clinical significance of effects across studies. Accordingly, the Discussion focuses on synthesising how associations between pain and functional outcomes have been described in the literature, highlighting recurring patterns and their potential clinical implications, while explicitly acknowledging the limitations in interpreting effect magnitude. This approach aligns with the objectives of a scoping review and supports clinically meaningful interpretation without overstating quantitative inference.

5. Although some limitations are acknowledged, the discussion would benefit from a more comprehensive consideration of potential biases inherent to the included study designs, including selection bias, performance bias, and outcome reporting bias, and how these may affect the conclusions.

Thank you for this comment. We have expanded the Discussion to provide a more comprehensive consideration of potential biases inherent to the included study designs, including selection bias, performance bias, and outcome reporting bias, and to clarify how these sources of bias may influence interpretation of the findings. Specifically, in the revised manuscript, we added “Several sources of bias inherent to the included study designs should be acknowledged. Many studies relied on convenience samples and cross-sectional designs, introducing potential selection bias and limiting causal inference. Variability in rehabilitation approaches, prosthetic components, and assessment protocols may also contribute to performance bias, while heterogeneity in outcome selection and reporting raises the possibility of outcome reporting bias. These factors should be considered when interpreting the findings of this review.”

6. The manuscript reports changes in patient-reported outcome measures but does not consistently contextualize these changes against established minimal clinically important differences (MCIDs). A dedicated discussion of whether observed improvements meet or exceed MCID thresholds is necessary to distinguish statistical from clinical relevance. The authors should cite and incorporate available MCID methodology and interpretation frameworks (DOI: 10.1002/jeo2.70568).

Thank you for this insightful comment. We agree that minimal clinically important differences (MCIDs) play a critical role in distinguishing statistical significance from clinical relevance. In response, we have incorporated a dedicated discussion in the revised manuscript highlighting the importance of MCID-based interpretation and have cited the recommended methodological framework (DOI: 10.1002/jeo2.70568). Specifically, in the revised manuscript, we proposed “To advance the field beyond descriptive associations and towards clinically actionable evidence, several priorities should be addressed. First, future studies should adopt integrated assessment protocols that combine validated pain measures, preferably multidimensional tools capturing pain intensity, interference, and activity dependence, with objective functional evaluation. Such integration would enable more direct linkage between pain profiles and movement adaptations and support interpretation of whether observed changes represent clinically meaningful improvement from a patient perspective, rather than statistical change alone, including consideration of established minimal clinically important difference (MCID) thresholds[143,144]. Future research may also

benefit from incorporating objective approaches to pain evaluation that are well established in other clinical populations but have not yet been applied in LLA research, including standardised laboratory-based experimental pain paradigms such as contact heat–evoked potentials and the cold pressor test[145], as complementary inputs to functional and biomechanical assessment. ”

However, as this study was conducted as a scoping review and the included literature exhibits substantial heterogeneity in outcome measures, reporting of change scores, and availability of validated MCID thresholds, it was not feasible to systematically determine whether reported improvements met or exceeded MCID values. Instead, we discuss MCID as an important interpretive framework and highlight the need for future studies to report outcomes in a manner that enables clinically meaningful interpretation. This approach aligns with the objectives of a scoping review while avoiding overinterpretation beyond the available evidence.
